# Learning shapes neural geometry in the primate prefrontal cortex

**DOI:** 10.1038/s41593-026-02333-w

**Published:** 2026-06-25

**Authors:** Michał J. Wójcik, Jake P. Stroud, Dante Wasmuht, Makoto Kusunoki, Mikiko Kadohisa, Mark J. Buckley, Rui Ponte Costa, Nicholas E. Myers, Laurence T. Hunt, John Duncan, Mark G. Stokes

**Affiliations:** 1 Centre for Neural Circuits and Behaviour, Department of Physiology, Anatomy and Genetics, University of Oxford, Oxford, UK; 2 Department of Experimental Psychology, University of Oxford, Oxford, UK; 3 Department of Engineering, University of Cambridge, Cambridge, UK; 4 Coherence Neuro Global, San Francisco, CA USA; 5 MRC Cognition and Brain Sciences Unit, University of Cambridge, Cambridge, UK; 6 Centre for Neurotechnology, Neuromodulation and Neurotherapeutics, University of Nottingham, Nottingham, UK; 7 Department of Psychiatry, University of Oxford, Oxford, UK

**Keywords:** Cognitive control, Neural decoding

## Abstract

The relationship between the geometry of neural representations and the task being performed is a central question in neuroscience. The primate prefrontal cortex (PFC) is a primary focus of inquiry, as it can encode information with geometries that either rely on past experience or are experience agnostic. One hypothesis is that PFC representations should evolve with learning, from a format that supports exploration of all possible task rules to a format that minimizes the encoding of task-irrelevant features and supports generalization. Here we test this idea by recording neural activity from the macaque PFC when learning a new rule (‘XOR rule’) from scratch. We show that PFC representations progress from being high dimensional, nonlinear and randomly mixed to low dimensional and rule selective. Upon generalizing the rule to new stimuli, these representations further evolve into an abstract, stimulus-invariant geometry. These findings reconcile previously conflicting accounts of PFC function by demonstrating how neural representations adapt across distinct stages of learning.

## Abstract (web summary)

Learning transforms prefrontal cortex activity from flexible, high-dimensional representations into compact, task-relevant and abstract codes, enabling efficient generalization of learned rules to new stimuli and contexts.

## Main

**Fig. 1:**
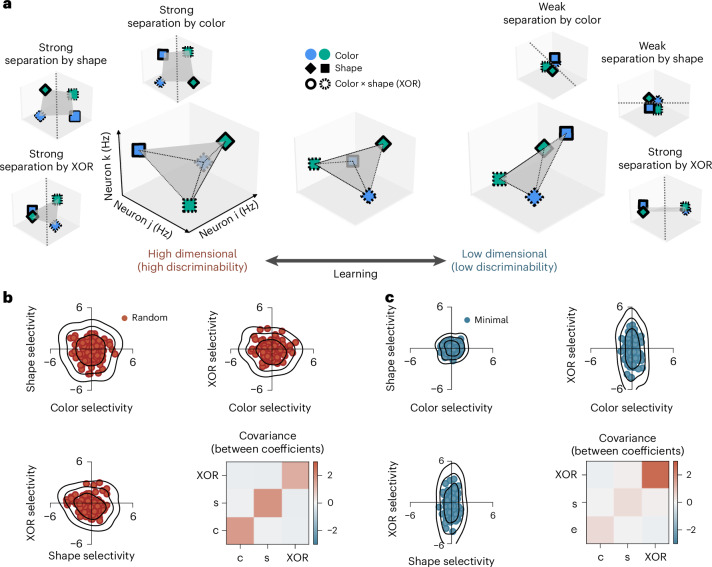
Potential effects of learning on neural geometry in the prefrontal cortex. Learning can reduce or expand neural dimensionality, changing how many linear decoding axes can be implemented on neural firing rates (discriminability). **a**, High-dimensional representations enable high discriminability. A high-dimensional regime allows the strong separation of all task features using three possible readout axes (left), whereas a low-dimensional representation only allows task-relevant features to be strongly separated (right). **b**, Each neuron can be represented as a point in the three-dimensional selectivity space spanned by color, shape and XOR (their interaction). In the random model, selectivity is distributed according to a spherical Gaussian distribution in this space (‘Generative models’); the covariance matrix is computed across the selectivity coefficients; zero-mean Gaussian noise ($\sigma \,=\,0.7$) was added to each selectivity coefficient to illustrate measurement bias under finite sampling. **c**, Analogous to **b**, but for the minimal model; neurons are strongly selective only for the XOR (interaction between color and shape), as this is the only feature that is necessary to solve the task. c, color; s, shape.

Two seemingly discrepant accounts propose that primate prefrontal cortex (PFC) neural activity should track either low-dimensional^1–7^ or high-dimensional^8–11^ representations of the environment. Traditionally, it has been proposed that PFC cells are tuned adaptively to task-relevant information, leading to low-dimensional neural activity^6^. This results in the population displaying structured selectivity patterns, as commonly observed after training on a cognitive task (Fig. 1a, low dimensional)^6^. A contrasting hypothesis suggests that the PFC may rely on high-dimensional, nonlinearly mixed representations of task features to support complex cognition (Fig. 1a, high dimensional)^8,9^. According to this notion, the PFC serves as a nonlinear kernel such that when a low-dimensional input is projected onto it, dimensionality expands, and a wide repertoire of responses can be generated^10,11^.

Recently, it has been proposed that the PFC can transition between high-dimensional and low-dimensional representations during learning to accommodate the changing demands of the environment^12–15^. For example, early in learning, high-dimensional representations may allow flexible exploration of all possible input–output mappings (‘contingencies’) to discriminate which task rules are currently relevant^8,9,11^. This is because a high-dimensional representation allows for a high number of linearly separable task features (Fig. 1a). Conversely, once an animal has learnt that only one set of contingencies is relevant, a low-dimensional representation may be used to encode task-relevant features more robustly^7,12–14^. Moreover, these low-dimensional representations may enable generalization to new contexts, as aligning new with old representations is likely easier when fewer dimensions must be considered^1,16^. In other words, different stages of learning impose different demands on the neural population. Learning could thus shape neural dimensionality and progressively push neural activity toward different solutions along the trade-off between discriminability and generalizability, that is, from a high-dimensional regime toward a low-dimensional regime^14,15^.

Here we tested this idea in two macaques that learned an exclusive-or (XOR) rule—a problem that can be solved by various representations, from low to high dimensional (Fig. 1a)^7^. Notably, we tracked how the dimensionality and geometry of PFC representations changed across multiple training sessions of an XOR rule that was entirely new to the animals at the start of recording (experiment 1) and during subsequent generalization of this rule to a new stimulus set (experiment 2). We used a classical conditioning paradigm in which the nonlinear combination of the features of two objects presented in succession (XOR) predicted the outcome of the trial. Notably, the animals were only required to fixate through both experiments^17–20^. Later, we also show that our results hold in a previously collected delayed match-to-sample task^21–23^.

Across two experiments, we found that, during early stages of learning, PFC activity was high dimensional, with individual neurons showing nonlinear and randomly mixed selectivity. As learning progressed, population activity became increasingly low dimensional, with structured selectivity emerging predominantly for task-relevant variables. When new combinations of stimuli were introduced, PFC representations reorganized such that new and familiar conditions aligned along a shared axis, enabling the re-use of a common neural code. These findings demonstrate that learning reshapes both the dimensionality and geometry of neural representations in the PFC, promoting low dimensional and abstract encoding when animals continuously engage in a single task structure.

## Results

### Generative models of nonlinear random and minimal selectivity

We first wanted to understand how the geometry of the neural representations could change over the course of learning. We thus explored the geometries produced by two generative models of neural selectivity with different discriminability–generalizability trade-offs^12,13^—(1) a high-dimensional geometry produced by nonlinear random mixed selectivity^8–11,24^ (high discriminability, low generalizability) and (2) a low-dimensional geometry produced by structured, minimal selectivity (low discriminability, high generalizability)^3,5,25–29^. The former is a well-established geometry (for example, inherent in reservoir computing models^10,11^), whereas the latter was inspired by work suggesting that PFC neurons flexibly adapt their selectivity to current task demands^6^. These models make distinct predictions about the distribution of selectivity to the task-relevant variables in a three-dimensional selectivity space (color, shape and their nonlinear interaction, that is, XOR). We refer to minimal or random selectivity when describing the distribution of selectivity and to low-dimensional or high-dimensional geometry when referring to the respective representations generated by these distributions (see Discussion for a detailed distinction between dimensionality and selectivity).

**Extended Data Fig. 1:**
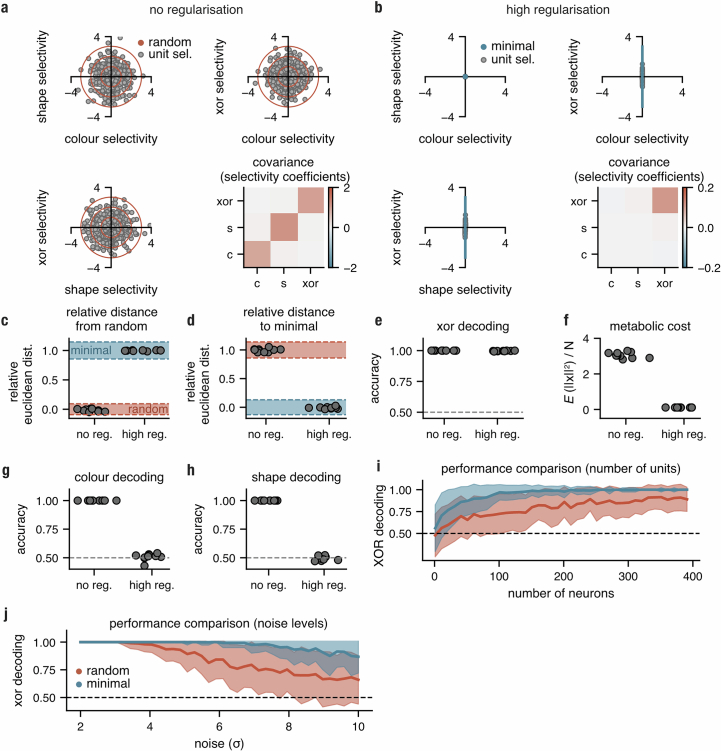
Optimized feedforward networks converge to the minimal XOR selectivity model. Twenty feedforward networks were trained (10 with high levels of regularization and 10 with no regularization) to perform the XOR task. **a**,**b**, Selectivity observed in no and high regularization networks, respectively; models of random (red ellipses) and minimal selectivity (blue ellipses) well approximated the observed selectivity. **c**,**d**, After training, low regularization networks converged on a random mixed selectivity regime and high regularization networks on a minimal XOR regime. **e**, Post-training XOR decoding (linear SVM) for both no and high regularization models. **f**, No regularization models exhibited substantially lower metabolic cost (Supplementary Note, equation (8)). **g**,**h**, Color and shape decoding (linear SVM) for no and high regularization models, respectively. **i**,**j**, Comparison of XOR decoding obtained from minimal and random generative models as a function of population size (**i**) and noise (σ) levels (**j**). Dashed gray line shows chance-level decoding; shaded areas correspond to $\pm 1{\rm{s}}.{\rm{d}}.$ of the mean. Source data

In selectivity space, each axis represents a unit’s response to one stimulus variable (for example, high shape selectivity = higher firing rate for square than diamond; Fig. 1b). Hypothetically, one could imagine different distributions of variable encoding within this selectivity space. The properties of these distributions are determined by a covariance matrix—the diagonal elements describe the strength of coding of each variable (variance), whereas the off-diagonal entries determine the strength of the relationship across variables (covariance). In our generative models, neural firing rates were simply constructed as a linear combination of task variables (color, shape and XOR inputs represented using one-hot encodings; ‘Generative models’) with a specified covariance matrix of selectivities to the task variables. In line with previous studies, the high-dimensional model was constructed by allowing selectivity to linear (color and shape) and nonlinear (XOR) features to be distributed randomly according to a spherical Gaussian distribution (Fig. 1b). In contrast, in the minimally structured XOR selectivity model, neurons were only strongly selective for the nonlinear interaction (that is, the XOR; Fig. 1c). One possible way of constructing such a model is considering biologically plausible limits on neural firing rates (minimizing net firing rate). In line with this, we derived this model mathematically and demonstrated that it minimizes total firing-rate activity while maximizing task performance (Supplementary Note). Consistent with this, we found that feedforward networks trained using backpropagation to perform the task while also minimizing a metabolic cost term converge to the minimal XOR selectivity model (Extended Data Fig. 1a–h; ‘Optimized feedforward networks’). Please note that alternative mechanisms, such as initialization or the presence of noise, could also be applied to learn a similar minimal selectivity model^3,30^. In line with prior accounts^12,16^, XOR decoding in the low-dimensional model required fewer units to implement a stable readout (Extended Data Fig. 1i) and was more robust to noise (Extended Data Fig. 1j).

**Extended Data Fig. 2:**
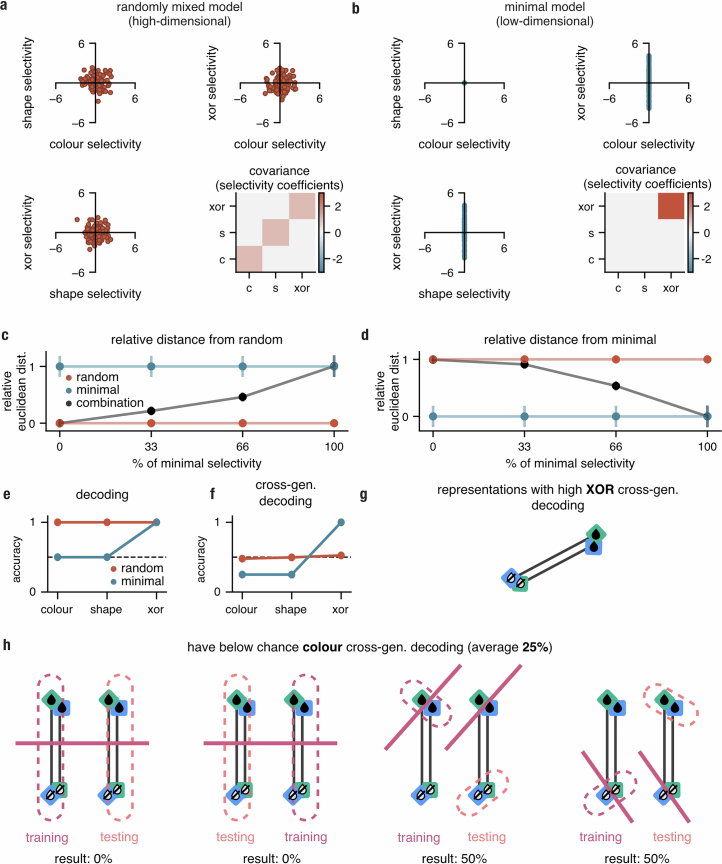
Predictions from random mixed and minimal selectivity models. **a**, Each neuron can be represented as a point in the 3-dimensional selectivity space spanned by color, shape, and XOR (their interaction). In the random mixed model, selectivity is distributed according to a spherical Gaussian distribution in this space (Generative models); the covariance matrix is computed between the selectivity coefficients. **b**, Analogous to **a** but for the minimal model; neurons are selective only for the XOR (interaction between color and shape), as this is the only feature that is necessary to solve the task. **c**, Relative distance between the covariance matrices of either the random (red), minimal (blue) or the model with varying proportions of minimal selectivity (black) to the random selectivity model. **d**, Same as panel **c**, but the distance is calculated relative to the minimal selectivity model. Red and blue error bars show standard deviations ($\pm 1{\rm{s}}.{\rm{d}}.$ over 1000 randomly drawn models; Measuring similarity between selectivity distributions) of the relative Euclidean distance between the covariance matrix of the random and the minimal model (with matched total variance to the data; standard deviation of random-to-minimal distance is too small to be visible; red) and relative Euclidean distance between the covariance matrix expected from two randomly drawn minimal models (blue); black error bars show the standard deviation of the relative distance between the surrogate covariance (with varying proportions of minimal selectivity) and random covariance ($\pm 1{\rm{s}}.{\rm{d}}.$ over 1000 random models). **e**, Mean (over 100 models) decoding of task variables for the random (red) and minimal (blue) models. Dashed gray line shows chance-level decoding. **f**, Mean (over 100 models) cross-generalized decoding of task variables for the random (red) and minimal (blue) models. Dashed gray line shows chance-level decoding. **g**,**h**, Relationship between high XOR cross-gen. decoding and below-chance color cross-gen. decoding. The clustering of all rewarded trials (XOR == true) and nonrewarded trials (XOR == false) on opposite sides of an axis (representing the abstract XOR) results in high cross-gen. decoding for the XOR (**g**). Consequently, color exhibits below-chance cross-gen. decoding. This occurs because some color decoding axes are inverted between training and testing splits, leading to 0% accuracy scores (**h**; left), while others assign a single class label to all examples when testing, resulting in a 50% score (**h**; right). The same argument holds for shape. Source data

We established a metric to measure whether neural activity is better described by the random or minimal model. We first fitted a linear model regression to surrogate data generated by both our generative models, in which task variables (color, shape and color × shape (XOR)) were used as predictors of each unit’s firing rate (‘Models’; for similar analysis, see refs. ^31,32^; Extended Data Fig. 2a,b). Then we measured the average within-model distance between two covariance matrices drawn from the random model (Extended Data Fig. 2c, red line; equation (4)) and the average between-model distance across the covariances drawn from the random and the minimal model (Extended Data Fig. 2d, blue line; equation (5)). To test whether our measure captures learning dynamics, we constructed four artificial populations with varying proportions of random and minimal selectivity. As the proportion of the minimal model in this mixed population increased, it became more dissimilar to the average random model and more similar to the average minimal model (Extended Data Fig. 2c, black line). A reflected version of these results held true when the minimal model was used as reference (Extended Data Fig. 2d).

Subsequently, to gain insight into the task geometries that these models generated, we used an established technique^8,9^ and trained linear decoders to decode all three task variables in both models. As previously suggested^8,9^, a randomly mixed selectivity model yielded a high-dimensional task representation, allowing for all variables, including the nonlinear XOR, to be decoded (Extended Data Fig. 2e, red, and Fig. 1a, far left). In contrast, for the minimal model, only the XOR combination of shape and color, and not shape or color independently, could be decoded (Extended Data Fig. 2e, blue, and Fig. 1a, far right). While both models can perform the task, we expected their representation of the XOR variable to differ fundamentally. On the one hand, the minimal model by design should represent the XOR in a format that generalizes over all other task variables. On the other hand, this is not guaranteed in the random model. We verified this intuition using cross-generalized decoding, a method in which a linear decoder is trained to decode a given task variable (for example, XOR = true versus XOR = false) on a given set of task conditions (for example, blue color) and tested on a different set of task conditions^16^ (for example, green color; ‘Cross-generalized decoding’). We found that, for the random model, cross-generalized decoding was at chance level for all task variables (Extended Data Fig. 2f, red). This is because the random model, by design, shows no reliable structure in its representation of variables and therefore these dimensions are represented randomly in relation to each other. In contrast, the minimal model displayed maximal cross-generalized decoding for the XOR variable (Extended Data Fig. 2f, blue), indicating that it can be decoded regardless of which set of task conditions the decoder is trained and tested on. This suggests that the minimal model is able to represent the XOR in a highly cross-generalizable format (Fig. 1a, far right). Consequently, the minimal model also shows below-chance cross-generalized decoding for color and shape (Extended Data Fig. 2g,h). Next, we directly compared neural data at each stage of learning to the selectivity (random versus minimal) and neural geometry (low dimensional versus high dimensional) generated by these models.

### Learning a single task reduces neural dimensionality in the prefrontal cortex

**Fig. 2:**
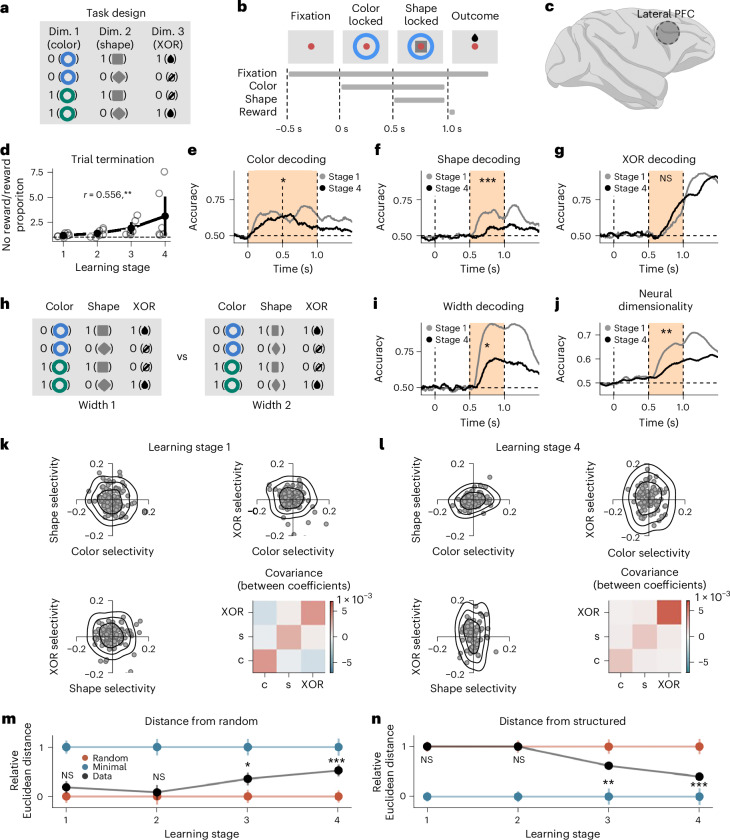
Neural representations in macaque PFC during learning of a single task. **a**, In experiment 1, animals were incentivized to combine two passively viewed task features (color and shape) in a nonlinear fashion (XOR). For example, blue + square and green + diamond combinations were rewarded, whereas blue + diamond and green + square were not. **b**, Timeline of task events in a single trial. **c**, Neural data were collected from the lateral surface of the prefrontal cortex in two macaque monkeys (for further details, see ‘Data acquisition and preprocessing’). The brain illustration in **c** was based on the NIMH Macaque Template v2 (ref. ^45^). **d**, The tendency of animals to terminate trials in the shape-locked trial period plotted as a ratio of termination numbers in not rewarded and rewarded trials (illustrated as a function of learning). Data are shown as mean ± 95% CI of session-level termination indices. Open circles represent individual sessions (*n* = 25 sessions from two animals); sessions were grouped into four learning stages; a one-sided permutation test was used. **e**–**g**, Time-resolved linear decoding in stage 1 (gray) and stage 4 (black) of color (**e**), shape (**f**) and XOR (**g**); the pale orange shaded areas denote the time windows in which permutation tests were conducted to assess differences between stage 1 and stage 4 decoding scores (in presentation order—P=0.029; P<0.001; P=0.813; one-sided tests); vertical three dashed lines show the onset of the color, shape and the outcome. **h**, Schematic of narrow and broad shape trials (width feature). This feature was not predictive of reward. **i**, Temporally resolved linear SVM decoding of width (P=0.032, one sided). **j**, Time-resolved neural dimensionality as measured by shattering dimensionality (all possible task dichotomies; P=0.01, one sided). **k**,**l**, Neural selectivities in learning stages 1 (**k**) and 4 (**l**) computed in the late shape-locked period ($\left({t}_{400\,\mathrm{ms}},{t}_{500\,\mathrm{ms}}\right)$, shape locked). Each point represents the selectivity of one neuron for each of the task variables (color, shape and XOR). All three possible pairs of the three axes are shown. The contour plots represent a kernel density estimate of the data, with each ring corresponding approximately to 1, 2 and 3 s.d. from the mean. Right, the covariance matrix of the selectivities computed from the data for stage 1 of learning (**k**) and stage 4 of learning (**l**). **m**, Relative Euclidean distance between the covariance matrix of selectivity coefficients from the data ($\left({t}_{400\,\mathrm{ms}},{t}_{500\,\mathrm{ms}}\right)$, shape locked) and the covariance matrix expected from random selectivity (with matched total variance) as a function of learning (‘Measuring similarity between selectivity distributions’). Red and blue error bars show mean ($\pm 1\;{\rm{s}}.{\rm{d}}.$ over 1,000 randomly drawn models) of the relative Euclidean distance between the covariance matrix of the random and minimal models (with matched total variance to the data) and the covariance matrix expected from random selectivity. One-sided, FWE-corrected permutation tests assessed whether observed selectivity was closer to random than expected by chance (stages 1 and 4—P=0.316, P=0.999, P=0.028, P<0.001); black error bars show s.d. of relative distance between the observed covariance and random covariance ($\pm 1\;{\rm{s}}.{\rm{d}}.$ over 1,000 random models). **n**, Same as **m**, but the relative distance of the observed covariance ($\left({t}_{400\mathrm{ms}},{t}_{500\mathrm{ms}}\right)$, shape locked) from the covariance matrix expected from minimal selectivity is shown (with matched total variance; stages 1 and 4—P=0.999, P=0.999, P=0.004, P<0.001; one-sided, FWE corrected). All *P* values were calculated from permutation tests (***P<0.01; **P<0.01; *P<0.05). NS, not significant; CI, confidence interval; FWE, family-wise error. Source data

In experiment 1, the animals were trained to combine a color stimulus (either blue or green) with a subsequently presented shape (either square or diamond) in a nonlinear fashion to predict the reward (the XOR between color and shape) outcome of the trial (Fig. 2a,b). Using a semichronic multielectrode system, we sequentially recorded 376 neurons from the lateral PFC across both macaques (Fig. 2c; ‘Data acquisition and preprocessing’). Moving electrodes between sessions ensured that a new sample of neurons was obtained in each session. Notably, to capture learning dynamics, we started recording from the first session in which the animals were exposed to the task. Experimental sessions were split into four learning stages for each animal separately. Data for each stage were combined across animals. A sliding-window approach was used to apply all available trials while ensuring an equal number of trials per learning stage; each stage comprised approximately equal numbers of training sessions (‘Data acquisition and preprocessing’). Selectivity analyses were run in the time window before the animals received feedback about the outcome of the trial (that is, reward; ‘Data acquisition and preprocessing’).

**Extended Data Fig. 3:**
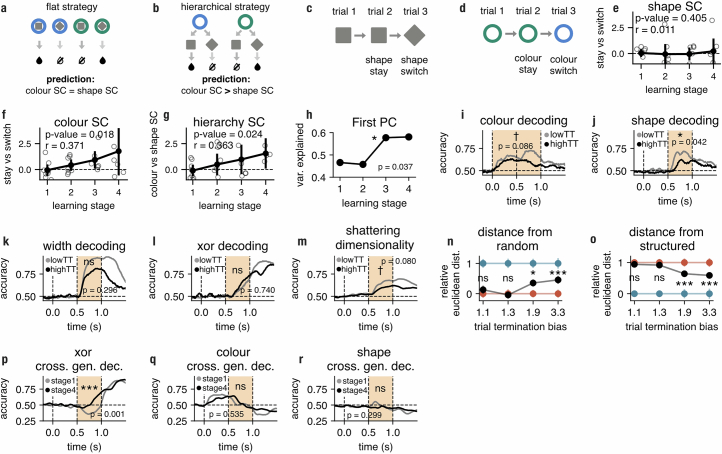
Decoding of task variables as a function of learning and behavior in experiment 1. **a**,**b**, The animals could adopt two possible strategies to learn the task: (1) memorizing every stimulus–outcome pairing, or (2) using a hierarchical strategy in which color served as a first-order policy cue, guiding subsequent context-dependent processing of shape. To differentiate between these strategies, color and shape switch costs were computed. **c**, Shape switch costs: trial termination rates were compared between trials in which the shape changed vs. remained the same on consecutive trials. **d**, Analogous illustration but for color switch costs. **e**–**g**, Trial termination switch costs for color, shape, and the difference between color switch costs and shape switch costs, plotted as a function of learning stage. Data are shown as mean ± 95% CI of session-level termination indices. Open circles represent individual sessions (n = 25 sessions from 2 animals); sessions were grouped into four learning stages; a one-sided permutation test was used. **h**, Variance explained (ratio) by the first principal component plotted as a function of learning (see PCA for details). **i**–**o**, Analogous to Fig. 2 but run on sessions sorted by proportion of adaptive trial termination (TT); statistical tests for decoding analyses were conducted on firing rates averaged over the time window indicated in pale orange; tests in **n** and **o** were FWE-corrected (distance to random, stages 1–4: p=0.999, p=0.999, p=0.032, p=0.008; distance to minimal, stages 1–4: p<0.999, p=0.999, p<0.001, p<0.001). **p**–**r**, Temporally resolved cross-generalized decoding of XOR cross. gen. dec. (**p**), color cross. gen. dec. (**r**), and shape cross. gen. dec. (**r**) in stage 1 and stage 4; the pale orange shaded areas indicate the time window in which statistical tests were run. All p-values were calculated from one-sided permutation tests (***, p<0.01; **, p<0.01; *, p<0.05; †, p<0.01; n.s., not significant). Source data

We first assessed learning behaviorally by examining the animals’ tendency to terminate nonrewarded trials before the potential reward onset (Fig. 2b, shape-locked period) by breaking fixation (Fig. 2d). This was quantified by calculating a trial termination index, that is, the ratio of terminated nonrewarded trials to terminated rewarded trials, which we tracked across different learning stages (‘Adaptive trial termination and switch costs’). Over the course of learning, the animals increasingly differentiated between rewarded and nonrewarded trials (r=0.56,P<0.01; Fig. 2d). We then investigated which learning strategy the animals adopted. One possibility is that they memorized each stimulus combination and its associated outcome (a ‘flat strategy’; Extended Data Fig. 3a). Alternatively, they may have used a hierarchical strategy, in which color served as a first-order policy cue guiding subsequent context-dependent processing of shape (Extended Data Fig. 3b). To distinguish across these strategies, we analyzed switch costs (‘Adaptive trial termination and switch costs’), comparing the animals’ ability to terminate nonrewarded trials following a change in color versus shape from the previous trial (Extended Data Fig. 3c,d). We found no evidence of shape-switch costs (r=0.01,P=0.405; Extended Data Fig. 3e), whereas color-switch costs increased over learning (r=0.37,P<0.05; Extended Data Fig. 3f) and were significantly greater than shape-switch costs (r=0.36,P<0.05; Extended Data Fig. 3g). These results suggest that animals increasingly relied on color as a higher-order cue, consistent with the adoption of a hierarchical learning strategy.

We then applied our linear decoding analyses to the neural recordings to establish whether the emergence of this behavioral strategy was accompanied by changes in the dimensionality of neural representations. At the beginning of training (learning stage 1, gray lines), the animals showed a high-dimensional geometry that allowed for color, shape and XOR to be decoded, reminiscent of the high-dimensional model (Fig. 2e–g). This was especially prominent in the period before the reward delivery.

Over the course of learning (stage 1 versus stage 4), we observed a reduction in color decoding (P<0.05, one sided) and shape decoding (P<0.001, one sided) but not XOR decoding (Fig. 2e–g, gray versus black lines). The shape stimuli also had a feature that was irrelevant for the prediction of the outcome—width (Fig. 2h). Similarly to color and shape, the decoding of this feature also decreased over learning (P<0.05, one sided; Fig. 2i). The reduction of color and shape coding as well as stable output-relevant feature (XOR) decoding was predicted by a transition from a high-dimensional to a low-dimensional model (Extended Data Fig. 2e, red versus blue).

We then explicitly tested whether the dimensionality of neural representations changed over learning (as measured with shattering dimensionality; for details, see ref. ^8^ and ‘Decoding’). A neural representation described by three binary input dimensions (color, shape and width) results in 35 dichotomies (division into two sets of four stimuli) that can be theoretically decoded. We found that the mean decoding accuracy of all dimensions decreased significantly over learning (P=0.01, one sided; Fig. 2j). Additionally, a principal component analysis (PCA) computed on condition averages revealed that the proportion of variance explained by the first principal component (PC) increased as a function of learning (${M}_{\mathrm{stage}\,1}=0.466\,\mathrm{versus}\,{M}_{\mathrm{stage}\,4}=0.581,\,P < 0.05$, one sided; Extended Data Fig. 3h; ‘PCA’).

We then tested whether changes to neural dimensionality were reflected in changes to selectivity. We fitted a linear regression to our data, just as we did for our generative models (Extended Data Fig. 2a,b), in which task variables (color, shape and color × shape (XOR)) were used as predictors of each neuron’s firing rate. We then examined how selectivity coefficients changed over learning (Fig. 2k, learning stage 1, and Fig. 2l, learning stage 4). We compared the covariance structure of these selectivity coefficients (Fig. 2k,l, bottom right) to the covariances obtained from the random selectivity model and minimal selectivity model (Extended Data Fig. 2a,b; covariance). At the beginning of learning (stage 1), PFC cells were randomly distributed in selectivity space resembling the high-dimensional model (P=0.316, one sided, Fig. 2m and P=0.999, one sided, Fig. 2n). However, in late learning (stage 4), selectivity diverged away from randomly mixed selectivity (P<0.001, one sided, Fig. 2m, compare black and red lines) and converged toward the minimal model (P<0.001, one sided; Fig. 2n, compare black and blue lines).

**Extended Data Fig. 4:**
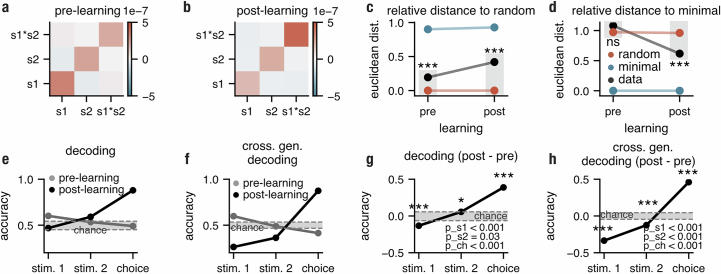
The re-analysis of an existing lPFC dataset. Same analysis methods were used as in Fig. 2 to test whether the PFC activity reported in ref. ^21^ converged on a minimal XOR model. **a**, The covariance matrix describing relations between the selectivity for stimulus 1, stimulus 2 and their interaction (XOR) in the prelearning phase of the experiment. **b**, Same as **a**, but for postlearning. **c**, Relative Euclidean distance between the covariance matrix of observed selectivity coefficients over learning and the covariance matrix expected from random selectivity (with matched total variance; pre, post: p<0.001, p<0.001; one-sided, FEW-corrected). **d**, Same as **c**, but the relative distance from the covariance matrix expected from minimal selectivity is shown (with matched total variance; pre, post: p=0.999, p<0.001; one-sided, FEW-corrected). **e**, Decoding of task variables for pre- and postlearning stages. **f**, Cross-generalized decoding of task variables plotted as a function of learning. **g**,**h**, Learning-induced accuracy differences in decoding and cross-generalized decoding, respectively. Shaded areas in **e**–**h** illustrate chance-level decoding obtained by shuffling trial labels (for details, see Statistical testing and reproducibility). All p-values were calculated from permutation tests (***, p < 0.01; **, p < 0.01; *, p < 0.05; n.s., not significant). The shaded gray regions in **e**–**h** represent the 95% nonrejection region of a permutation-based null distribution, computed by shuffling neuron-to-label assignments within each learning stage (**g**) and neuron-to-learning stage assignment across stages (**h**). Source data

We replicated a similar pattern of decoding and selectivity results on data sorted by the trial termination index. Here the behavioral measure served as a performance indicator, and sessions were sorted separately for each animal before being pooled into four pseudopopulations across animals (‘Adaptive trial termination and switch costs’; Extended Data Fig. 3i–o). Furthermore, we also performed a re-analysis of an existing dataset^21–23^ in which recordings were taken from primate ventral and dorsolateral PFC before and after learning a delayed match-to-sample task that was similar in structure to ours (‘Existing lPFC dataset’; Extended Data Fig. 4). We found that learning again pushed neural activity in the PFC toward a minimal regime.

Our findings indicate that neural activity in the PFC shifts between two distinct selectivity regimes as learning progresses. Initially, the PFC maximally expanded the representational space by encoding all available variables. Subsequently, after a combination of task variables that predicted the trial’s outcome was identified, neural activity became increasingly low dimensional. Such changes to neural dimensionality can be associated with simultaneous changes in neural geometry, potentially supporting more abstract representations (Extended Data Fig. 2f). To test this, we used cross-generalized decoding and, consistent with our generative models, observed a strong increase in cross-generalized XOR decoding across learning (P<0.001, one sided; Extended Data Fig. 3p). Although this effect is consistent with abstraction, it could also reflect motor-preparation or reward-prediction signals injected into the PFC in the rewarded XOR condition (XOR == true). To directly assess whether an abstract, task-relevant dimension emerges independently of such signals, we conducted experiment 2, introducing a new task instance that preserved the same underlying structure.

### Learning a single task structure promotes abstract neural geometry in the PFC

**Fig. 3:**
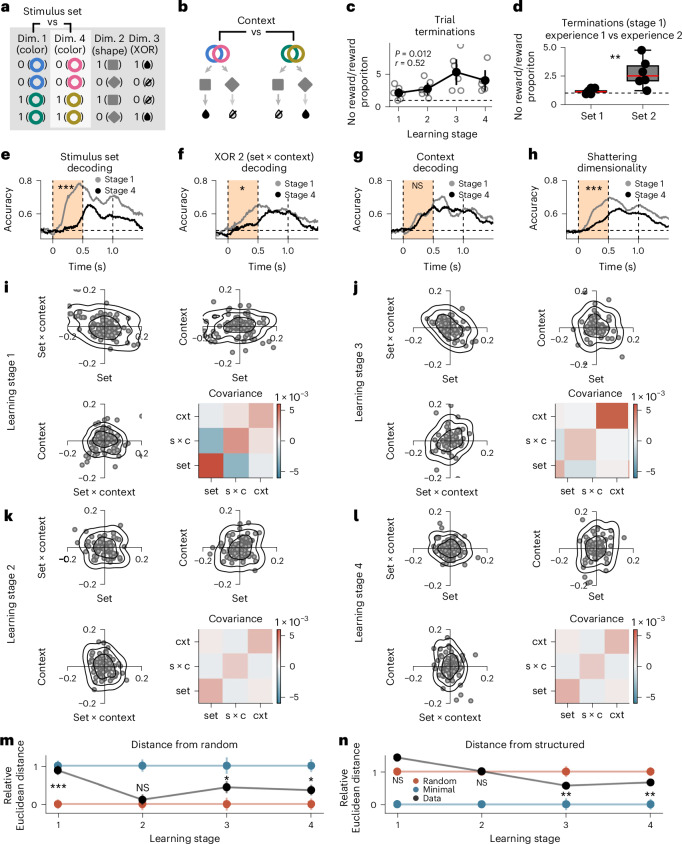
Neural representations in macaque PFC during learning of two instances of the same task structure. **a**,**b**, New colors (pink and khaki, stimulus set 2) were introduced in experiment 2, sharing the same shape–reward mapping as the learned colors (blue and green, stimulus set 1), shown as a factorisation of stimulus dimensions (**a**) and as a decision tree defining context as the colour pair gating the shape-reward mapping (**b**). **c**, Analogous to Fig. 2d, but computed for stimulus set 2 trials in experiment 2. Data are shown as mean ± 95% CI of session-level termination indices. Open circles represent individual sessions (*n* = 25 sessions from two animals; biological replicates at the session level); sessions were grouped into four learning stages. **d**, Comparison of the trial terminations observed in the first three sessions (per animal) in experiment 1 (when only stimulus set 1 was presented) and three first sessions (per animal) in experiment 2 for stimuli set 2 trials only (P=0.003; one sided). Box plots show the median (red line), IQR (box) and 1.5× IQR whiskers. Individual data points represent single recording sessions. **e**–**g**, Temporally resolved linear SVM decoding stimulus set (**e**), XOR 2 (set × context; **f**) and context (**g**; P<0.001; P=0.023; P=0.383; one-sided tests); the pale orange shaded areas denote the time windows in which permutation tests were conducted to assess differences between stage 1 and stage 4 decoding scores. Horizontal dotted lines represent chance-level decoding, whereas vertical dotted lines indicate the onset of the color, shape and the trial outcome. **h**, Time-resolved neural dimensionality as measured by shattering dimensionality (mean over all three possible task dichotomies in the color-locked period, that is, mean over **e**–**g**) in stage 1 and stage 4 (P=0.001; one sided). **i**–**l**, Neural selectivities in learning stages 1 (**i**), 2 (**k**), 3 (**j**) and 4 (**l**) computed in the color-locked period ($\left({t}_{200\,\mathrm{ms}},{t}_{500\,\mathrm{ms}}\right)$). Each point represents the selectivity of one neuron for each of the task variables (set, XOR 2 and context). All three possible pairs of the three axes are shown. The contour plots represent a kernel density estimate of the data, with each ring corresponding approximately to 1, 2 and 3 s.d. from the mean. Right, the covariance matrix of the selectivities. **m**,**n**, Analogous to Fig. 2m,n; compares set, XOR 2 and context selectivities ($\left({t}_{200\,\mathrm{ms}},{t}_{500\,\mathrm{ms}}\right)$, color locked) to idealized random and minimal models as a function of learning (distance to random, stages 1 and 4—P<0.001, P=0.42, P=0.012, P=0.028; distance to minimal, stages 1 and 4—P<0.999, P=0.999, P=0.004, P=0.008; one-sided, FWE corrected). All *P* values were calculated from permutation tests (***P<0.01; *P<0.05). IQR, interquartile range. Source data

**Extended Data Fig. 5:**
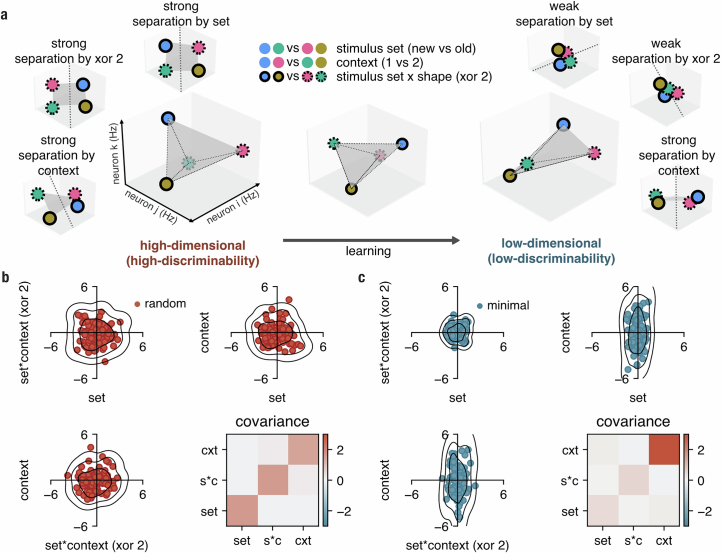
Hypothesized effects of learning on neural geometry and selectivity in the prefrontal cortex in experiment 2. As in Fig. 1, learning is depicted as reducing neural dimensionality, changing how many strong linear decoding axes can be implemented on neural firing rates (discriminability). **a**, A high-dimensional regime allows the strong separation of all task features using three possible readout axes (left), whereas a low-dimensional representation only allows task-relevant features to be strongly separated (right). **b**, Each neuron can be represented as a point in the 3-dimensional selectivity space spanned by stimulus set, stimulus set × context (XOR 2), and context (Fig. 3b). In the random model, selectivity is distributed according to a spherical Gaussian distribution in this space (Generative models); the covariance matrix is computed between the selectivity coefficients; zero-mean Gaussian noise (σ=0.7) was added to each selectivity coefficient to illustrate measurement bias under finite sampling. **c**, Analogous to **b**, but for the minimal model; neurons are strongly selective only for the context, as this is the only feature that is necessary to solve the task. Source data

In experiment 2, we introduced a new color pair (‘stimulus set’; Fig. 3a) that followed the same shape–outcome associations as the previous color pair (‘context’; Fig. 3b). Similar to experiment 1, we recorded neural activity from the very first session in which the animals were exposed to the new stimulus set and divided the experimental sessions into four distinct learning stages. To explore learning-induced changes to neural dimensionality, we again used linear decoding. We then compared these metrics to changes in the structure of neural selectivities. During the color-locked period, PFC activity could now represent three distinct variables (Extended Data Fig. 5a)—context (that is, the color indicating the relevant shape–outcome rule), stimulus set (set 1 versus set 2) and the nonlinear interaction between context and stimulus set (XOR 2). Using these three variables, we constructed selectivity models analogous to those in Fig. 2k,l (Extended Data Fig. 5b,c). Critically, in this design, context was the only task-relevant variable, whereas both the stimulus set and its nonlinear interaction with context (XOR 2) were irrelevant. We predicted that, as in experiment 1, PFC activity would transition from high dimensional and nonlinearly mixed to low dimensional and structured, with cells becoming increasingly selective for the task-relevant variable (Extended Data Fig. 5a). Additionally, randomly interleaving stimulus set 1 trials and stimulus set 2 trials allowed us to test whether a shared neural representation would be used for both stimulus sets. Notably, none of the analyses performed on activity during the color-locked period were confounded by motor preparation or reward prediction, as the shape information required to predict reward had not yet been presented.

We first examined the propensity of animals to terminate trials when the new stimulus set indicated a lack of reward, similar to the patterns observed in experiment 1. Over the course of learning with stimulus set 2, animals increasingly terminated nonrewarded trials more frequently than rewarded ones (r=0.52,P<0.05; Fig. 3c). Additionally, a facilitation effect was observed when comparing the first three sessions of experiment 2 (stimulus set 2 only) to the first three sessions of experiment 1 (stimulus set 1). Specifically, animals demonstrated significantly more adaptive trial termination early in the learning process with stimulus set 2 compared to stimulus set 1 (P<0.01; Fig. 3d). This early behavioral benefit may be attributed to the usage of the previously acquired task representation as a scaffold. Subsequently, we thus investigated how the neural representations of both stimulus sets interacted throughout the learning process.

**Extended Data Fig. 6:**
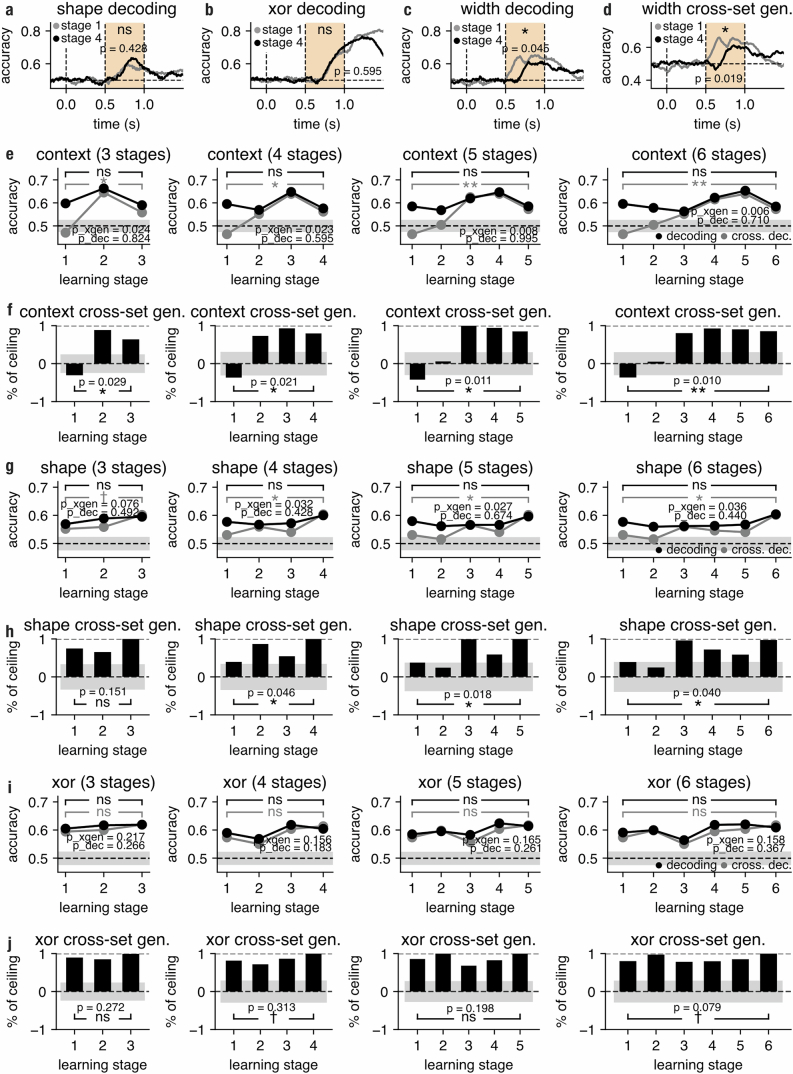
Decoding and selectivity results in experiment 2. **a**–**c**, Temporally resolved linear SVM decoding for shape, XOR and width; the pale orange shaded areas indicate the time window in which statistical tests were run. Horizontal dotted lines represent chance-level decoding, whereas vertical dotted lines indicate the onset of the color, shape and the trial outcome. **d**, Temporally resolved cross-generalized decoding of width (trained on set 1 and tested on set 2 (and vice versa)). **e**, Context cross-set generalization results remain consistent across different learning discretizations; analogs to Fig. 4d computed for 3, 4 (original), 5 and 6 learning stages (see Data acquisition and preprocessing for details). **f**, Cross-set context generalization normalized by linear context decoding (cf. **e**, black vs gray lines). Cross-set generalization performance is expressed as a proportion of linear context decoding (ceiling) across learning stages. Positive values indicate geometries in which color 1 vs color 2 generalizes to color 3 vs color 4, whereas negative values indicate the flipped geometry (color 1 vs color 2 generalizes to color 4 vs color 3). Gray-shaded areas indicate the null distribution obtained by shuffling trial labels. **g**,**h**, Analogous to **e** and **f**, but computed for shape cross-set generalization. **i**,**j**, Analogous to **e** and **f**, but computed for XOR cross-set generalization. All p-values were calculated from one-sided permutation tests **, p<0.01; *, p<0.05; †, p<0.01; n.s., not significant). The shaded gray regions in **e**–**j** represent the 95% nonrejection region of a permutation-based null distribution for cross-set generalization, computed by shuffling neuron-to-label assignments within each learning stage. The dashed line indicates the null distribution mean (chance level). Source data

Similarly to experiment 1, we hypothesized that the PFC would strongly represent all three variables—stimulus set, context and XOR 2 (that is, context × stimulus set)—at the beginning of learning, consistent with a high-dimensional coding regime (Extended Data Fig. 5a, left), and would then progressively transition to a low-dimensional representation in which most neurons are selective for context, the only task-relevant variable (Extended Data Fig. 5b, right). To test this hypothesis, we first used linear decoding (Fig. 3e–g). We found that both stimulus set decoding (P<0.001, one sided) and XOR 2 decoding (P<0.05, one sided) significantly decreased over learning, whereas context decoding remained stable across learning stages, mirroring the decoding results from experiment 1. Furthermore, we observed a significant reduction in shattering dimensionality during the color-locked period as learning progressed (P=0.001, one sided). No learning-related changes were detected for shape or XOR representations (Extended Data Fig. 6a,b). Notably, width decoding and geometry further declined with learning in experiment 2 (P<0.05, one sided; Extended Data Fig. 6c,d).

To determine whether these dimensionality changes were reflected in the structure of neural selectivity, we analyzed selectivity for the three task variables (context, set and XOR 2) at each learning stage and compared the population profiles to idealized random and minimal models (Extended Data Fig. 5b,c). In stage 1, neural selectivity significantly diverged from the random model and was inconsistent with the minimal model (P<0.001, one sided; Fig. 3m), driven by a strong preference for the stimulus set variable (stage 1; Fig. 3i). We interpret this as a novelty effect, with different neural responses for new versus familiar stimuli. In stage 2 (Fig. 3k), PFC selectivity shifted away from this initial set-selective regime and converged toward a profile consistent with randomly mixed selectivity (P>0.05, one sided, stage 2; Fig. 3m). From there, it gradually transitioned toward the minimal regime in stages 3 and 4 (Fig. 3j,l), reflecting increasingly structured and task-relevant coding ($\mathrm{stage}\,3,\,P < 0.05$, one sided, $\mathrm{stage}\,4,\,P < 0.05$, one sided, Fig. 3m; $\mathrm{stage}\,3,\,P < 0.01$, one sided, $\mathrm{stage}\,4,\,P < 0.01$, one sided, Fig. 3n). Together with experiment 1, these results identify random mixed selectivity as a critical stage in early learning, even when new information must first be integrated with existing representations. Subsequently, whether learning a completely new task (experiment 1) or a task related to prior knowledge (experiment 2), the PFC progressively converged toward minimal selectivity.

**Fig. 4:**
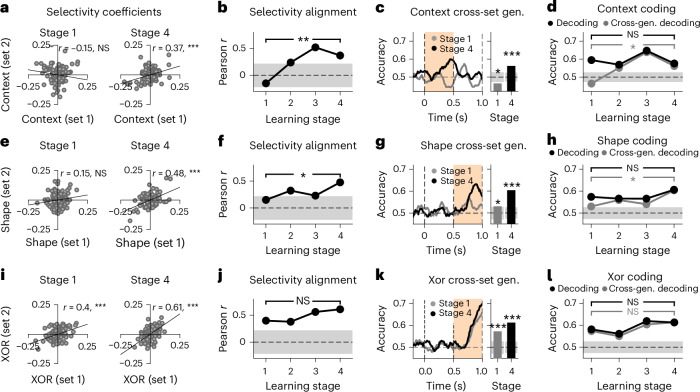
The PFC aligns new and old task representations as a function of learning. **a**, Selectivity of PFC neurons in stage 1 and stage 4 for context in set 1 and set 2 in the color-locked period. The line of best fit is shown in black (stage 1, P=0.122; stage 4, P<0.001; two-sided tests). **b**, Correlation between selectivity coefficients (computed in the color-locked period $\left({t}_{0\,\mathrm{ms}},{t}_{500\,\mathrm{ms}}\right)$) for context (context 1 versus context 2) in stimulus set 1 and stimulus set 2 in the late color-locked period plotted as a function of learning (P=0.007, one sided; cf. **a**). The shaded gray region indicates the mean ± 2 s.d. of the permutation-based null distribution, generated by shuffling neuron identities. **c**, Temporally resolved cross-generalized decoding of context (trained on set 1 and tested on set 2, and vice versa); pale orange shading indicates when the decoding and selectivity analyses in **a**–**d** were performed. Horizontal dotted lines represent chance-level decoding, whereas vertical dotted lines indicate the onset of the color, shape and the trial outcome. The bar plot represents the average cross-set generalization score of stage 1 and stage 4 compared against a null distribution obtained after shuffling trial labels (gray-shaded areas; stage 1, P=0.014; stage 4, P<0.001; one-sided tests). **d**, Decoding (black) and cross-generalized decoding (gray) of context as a function of learning in the color-locked period (pale orange area in **c**; decoding—P=0.595, two sided; cross-generalized decoding—P=0.023, one sided). **e**–**l**, Analogous to **a**–**d**, but for shape and XOR coding and selectivity. Test outcomes for shape in presentation order—stage 1, P=0.104; stage 4, P<0.001 (two sided; **e**); P=0.023 (one sided; **f**); stage 1, P=0.035; stage 4, P<0.001 (one sided; **g**); decoding—P=0.428 two sided); cross-generalized decoding—P=0.032 one sided; **h**). Test outcomes for XOR in presentation order—stage 1, P<0.001; stage 4, P<0.001 (two sided; **i**); P=0.133 (one sided; **j**); stage 1, P<0.001; stage 4, P<0.001 (one sided; **k**); decoding—P=0.183 (two sided); cross-generalized decoding, *P* = 0.156 (one sided; **l**). All *P* values were calculated from permutation tests (***P < 0.01; *P < 0.05). The shaded gray regions in **c**, **d**, **g**, **h**, **k** and **l** represent the 95% nonrejection region of a permutation-based null distribution for cross-set generalization, computed by shuffling neuron-to-label assignments within each learning stage. The dashed line indicates the null distribution mean (chance level). gen., generalization. Source data

We then examined whether the observed changes in dimensionality were accompanied by changes in neural geometry. Specifically, we assessed how task variables—context, shape and XOR—were coded across stimulus sets. For example, we asked whether the decision boundary separating the two contexts in stimulus set 1 (blue versus green) was the same as that in stimulus set 2 (pink versus khaki; Fig. 3b). We found that at the beginning of learning (stage 1) PFC cells that were selective to context in stimulus set 1 tended to be not selective to context in stimulus 2 (r=−0.15,P>0.05, one sided; Fig. 4a, left). After learning, in stage 4, the same neurons showed context selectivity that generalized across stimulus sets (r=0.37,P<0.001, one sided; Fig. 4a, right). Consistent with this, correlations between single-neuron context selectivity across color pairs increased progressively over learning (P<0.01, one sided; Fig. 4b). These changes at the single-neuron level were mirrored by a reorganization of population geometry. To quantify this, we trained a linear support vector machine (SVM) to decode context in stimulus set 1 and evaluated its performance on stimulus set 2, and vice versa (‘Cross-stimulus set generalization’; Fig. 4c,d). At the end of learning (stage 4), context decoding robustly generalized across stimulus sets, indicating that a common decision boundary was used regardless of the stimulus set presented (P<0.001, one sided; Fig. 4c, right). This geometry emerged with learning, reflected in an increase in cross-set generalized decoding from stage 1 to stage 4 (P<0.05, one sided; Fig. 4d, gray, ‘cross-decoding’). Please note that context was equally decodable in both early and late learning, indicating that the observed change in geometry was not driven by a change in the presence or absence of context information (P>0.05, two sided; Fig. 4d, black, ‘decoding’). These generalization effects were robust to how learning was discretized into stages (Extended Data Fig. 6e). Moreover, context cross-set generalization reached the ceiling level of simple context decoding after learning (Extended Data Fig. 6f), indicating that an abstract, stimulus-invariant geometry came to dominate the neural representation of context.

Stimulus set-invariant representations were also observed for shape and XOR. For shape, PFC neurons showed no significant alignment of selectivity across stimulus sets at stage 1 (r=0.15,P>0.05, one sided) but showed significant alignment after learning at stage 4 (r=0.48,P<0.001, one sided; Fig. 4e). This increase in correlation was learning dependent (P<0.05, one sided; Fig. 4f). Cross-set generalized decoding of shape was significant both early (stage 1; P<0.05, one sided) and late (stage 4; P<0.001, one sided; Fig. 4g). Although shape decoding did not change with learning (P>0.05, two sided; Fig. 4h, black), cross-set generalized shape decoding increased over learning (P<0.05, one sided; Fig. 4h, gray). These effects were robust across learning discretizations (Extended Data Fig. 6g). As for context, cross-stimulus-set generalization approached the ceiling of simple shape decoding after learning, indicating dominance of a stimulus set-invariant representational geometry (Extended Data Fig. 6h). For XOR, selectivity alignment and cross-set generalized decoding were already high at stage 1 and remained high at stage 4 (Fig. 4i–k), with learning providing no further improvement in these measures (Fig. 4j,l). These results were robust across learning discretizations (Extended Data Fig. 6i), and cross-set generalized XOR decoding remained near ceiling throughout learning (Extended Data Fig. 6j).

Together, these results show that even when new information fits an existing task schema, the PFC traverses a representational trajectory from high-dimensional, mixed coding toward low-dimensional, task-relevant abstraction. Critically, abstraction emerges through progressive alignment of neural geometry across stimulus sets, with some variables (XOR) generalizing almost instantly, while others (context, shape) require representational reorganization.

## Discussion

The prefrontal cortex has the capacity to generate both low-dimensional and high-dimensional representations, each of which presents a unique trade-off between generalizability and discriminability. However, the conditions under which each regime is used currently remain unclear. Our study investigated how the dimensionality and geometry of neural activity changed over learning. We observed that, as learning progressed, neural activity in the PFC transitioned from being high dimensional with high discriminability to being low dimensional and abstract. This transition in the representational strategy was accompanied by a change in patterns of single-cell selectivity, from random and nonlinearly mixed toward minimal and structured. The structured representations that emerged during learning then supported the generalization of the learned rule to a new stimulus set^18,20^.

We found that the PFC transitioned from a high-dimensional to a low-dimensional regime over multiple days of exposure to a complex task. This corroborates the findings mentioned in ref. ^5^, which showed that neural activity covaried with behaviorally relevant variables, thus occupying a low-dimensional manifold. On the other hand, some studies have suggested that an increase in neural dimensionality is predictive of performance^8^. It is possible that the structure of the task and training provides an explanation for these contrasting findings^14^. In our study, recordings were initiated from the onset of task training, spanning a period of 5 weeks, with animals experiencing the entire task structure from the first session. In contrast, many other investigations into the primate PFC’s involvement in complex cognitive tasks train animals in a fashion that decomposes the task into multiple subcomponents and either builds up task knowledge across training^8^ or presents them in a serial (block-wise) manner^16,33^. Additionally, whereas in our study one source of information (width) was always irrelevant, in other studies information becomes periodically relevant and irrelevant across multiple blocks, which may promote encoding of currently irrelevant information^32^. These training differences may promote high-dimensional information encoding that favors discriminability over abstraction.

The acquisition of a low-dimensional representation after the learning of a single XOR rule raises the question of which regime the PFC might adopt when confronted with more complex tasks. Although XOR operations necessitate nonlinear integration and abstraction from sensory input, they can be reduced to simple stimulus–response pairings once the rule has been learned or when a memory-based strategy is used. However, some tasks are more difficult to decompose, as they require switching between multiple orthogonal and conflicting subtasks. It has been suggested^14,15^ that, in conditions requiring the performance of multiple tasks in series, a high-dimensional representation could be used by the PFC to maximize flexibility and to prevent interference.

Our analytical approach aligns with a growing body of work that emphasizes the connection between population structure and neural coding^34^. Specifically, we investigated how diverse forms of single-cell selectivity contribute to the geometry and dimensionality of population-level representations. Our results reveal a negative correlation between neural dimensionality and the emergence of structured selectivity. However, the direction of this relationship may be more nuanced and task dependent. For instance, a highly structured population with pure selectivity for multiple variables shows higher dimensionality than a randomly mixed population responding to fewer variables, even if the latter is nonlinear.

Our findings suggest that the PFC could use a multiphase learning strategy, involving distinct temporal dynamics. Initially, new tasks could be solved through flexible, reservoir-like dynamics, bypassing the need for immediate synaptic plasticity. As training progresses over longer timescales, the PFC could gradually refine its local connectivity, optimizing for performance. This dual-phase approach enables both rapid adaptation and efficient resource allocation, echoing models of the cerebellum–motor cortex interactions, where the cerebellum rapidly drives cortical activity through input control^35^. Similarly, an external region could modulate the PFC’s activity on shorter timescales, enabling flexible high-dimensional representations. Over time, the PFC’s intrinsic circuitry would consolidate these representations and assume direct task control. Future research could explore the geometry of task representations acquired at different learning stages and the critical role of synaptic plasticity in this process.

The implicit assumption in many experimental paradigms is that the animals are presented with tasks as tabula rasa, devoid of prior knowledge or training. However, it is unlikely that the animal’s entire experimental history, including life experience, is irrelevant to a given task. Our second experiment allowed us to address this issue and explicitly explore the interactions between already learned and new information. In line with previous predictions^12,16^, we found that, when a new task instance is added, both the new and old instances were rapidly aligned to common axes and sensory differences between them were collapsed. Notably, different task motifs showed distinct generalization timescales—the XOR representation generalized early in learning, while the context motif required weeks of training to generalize. This disparity likely reflects differences in their structural composition. The XOR rule’s use of identical shapes across tasks likely facilitated rapid alignment, leveraging existing neural encoding schemes. In contrast, the context motif’s new colors necessitated additional encoding and adaptation in the prefrontal cortex, slowing generalization. This suggests that PFC’s representational alignment is modulated by the degree of overlap between prior and new stimuli. Shared features could thus promote efficient transfer of learned representations, while new features could impose additional encoding demands.

It is perhaps surprising that, given the key role of the PFC in the development and acquisition of structured knowledge, only a few studies have investigated how the structure of PFC representations changes during several training days of an entirely new task^4,36^. By tracking changes in neural activity across learning, it is possible to identify the biological principles that are required to produce representations supporting higher cognitive functions^37^. Future experiments should extend this paradigm to track changes in learning even more complex and naturalistic tasks^38^; those that have a compositional structure^39,40^; the influence of different learning curricula^41^; and how these representations change within the same individual neurons as opposed to pseudopopulations^42–44^.

## Methods

### Data and task

#### Animals and task

Two adult male rhesus macaques (*Macaca mulatta*), monkey 1 and monkey 2, were trained in this study (~14 kg each). The experiments were conducted in line with the Animals (Scientific Procedures) Act 1986 of the United Kingdom and licensed by a Home Office Project License obtained after review by the Animal Care and Ethical Review Committee at Oxford University. The procedures followed the standards set out in the European Community for the care and use of laboratory animals (EUVD, European Union directive 86/609/EEC). The animals were seated in a sound-attenuated and lighting-attenuated experimental booth. Their heads were restrained and faced a 19-inch screen. The center of the screen was aligned with a neutral eye position. The animals performed a passive object-association task (Fig. 2a,b). Notably, the animals were accustomed to an experimental setting but had no previous exposure to the task or stimuli introduced in this protocol. Neural recordings were collected from the first session, as one of the main aims of the study was to capture learning dynamics. In the first experiment, the animals were presented with a color and a shape, a nonlinear combination of which predicted reward (Fig. 2a,b). In experiment 2, a second set of stimuli was additionally introduced to test whether the rule learned in the first experiment cross-generalized to the new sensory domain (Fig. 3a). The colors used in the colored circles were designed in the CIELab color space^46^. The luminance (*L*) parameter was kept constant, ensuring that the stimuli were approximately isoluminant; parameters *a* and *b* varied with regard to valence but not value, resulting in a circular color representation^46^. As colors were randomly assigned to conditions for each animal, this circular representation ensured that, regardless of which color pair was assigned to which XOR mapping, the initial color similarity/dissimilarity within the color pair was kept constant. Additionally, in both experiments, the second object had two features—one relevant for reward prediction (Fig. 2a, shape) and one irrelevant (Fig. 2h, width, and, for the duration and sequence in which stimuli were presented, see Fig. 2b). The trial sequence was randomized. All trials with fixation errors were excluded. The dataset contained on average 237.9 (${\rm{s.}}{\rm{d}}.=23.9$) and 104.8 (${\rm{s.}}{\rm{d}}.=2.3$) trials for each of the 8 conditions in experiment 1, and 101.0 (${\rm{s}}.{\rm{d}}.=18.6$) and 54 (${\rm{s}}{.}{\rm{d}}{.}=1.1$) trials for each of the 16 conditions in experiment 2, for monkey 1 and monkey 2, respectively.

#### Data acquisition and preprocessing

**Extended Data Fig. 7:**
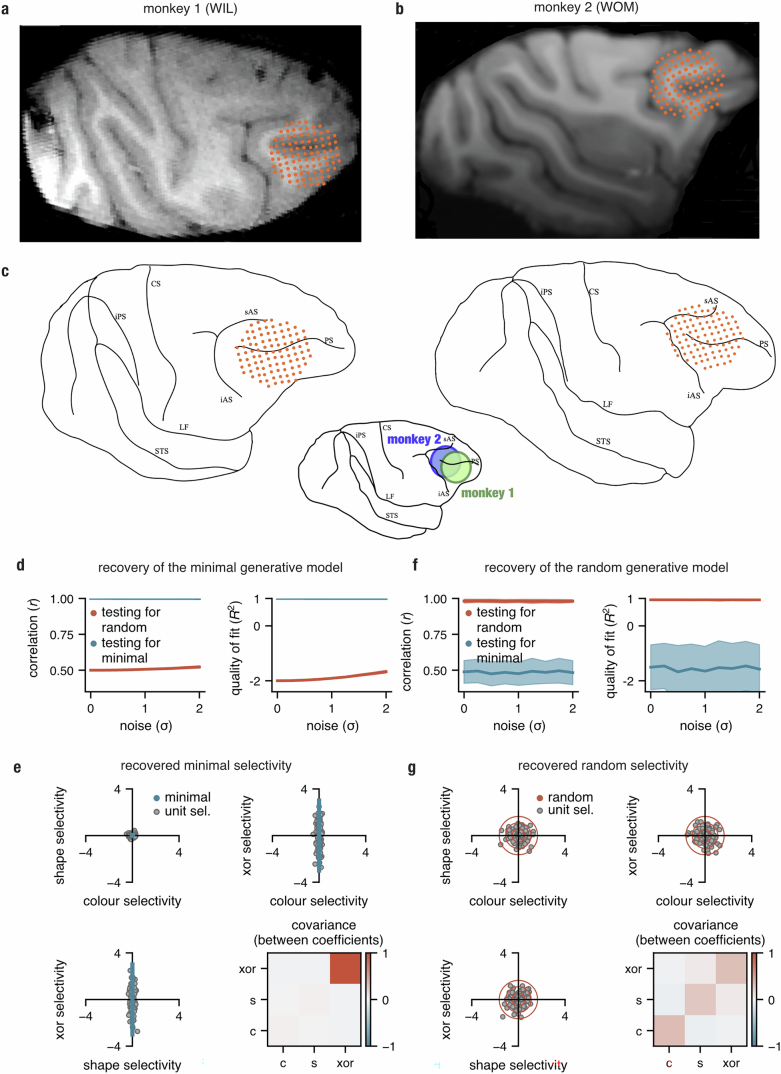
Neural recording locations and simulations of selectivity parameter recovery. **a**–**c**, Electrode locations in monkey 1 (**a**) and monkey 2 (**b**) and their comparison (**c**). **d**, Linear regression recovers underlying generative models. Pearson correlation and R^2^ computed between the covariance matrix of recovered selectivity and true underlying selectivity (when minimal generative model was used) for different levels of noise. **e**, Selectivity coefficients obtained after running a linear regression plotted for each unit in selectivity space (for ${\rm{\sigma }}=2$); minimal model overlaid in blue; covariance matrix computed between the recovered selectivity coefficients. **f**,**g**, Analogous to **d** and **e**, but when the random model was used to generate data; random model overlaid in red. Shaded areas in **a** and **c** indicate the mean $\pm 1{s}.d.$ computed over 100 different initializations. Source data

Before the start of the experimental protocol, a titanium head holder with two recording chambers was placed and fixed with stainless steel screws in each animal. The frontal recording chambers were implanted over the lateral prefrontal cortex (lPFC) of the right hemisphere in both animals. Data from a second chamber targeting inferotemporal cortex in the right hemisphere are not considered here. A craniotomy was made beneath each chamber to enable electrophysiological recording. Recording locations for each animal are shown in Extended Data Fig. 7a–c. Surgical procedures were carried out under general anesthesia and were aseptic. A semichronic microdrive system (SC-96, Gray Matter Research) with 1.5 mm interelectrode spacing, interfaced to a multichannel data acquisition system (Cerebus System, Blackrock Microsystems), was used for frontal recordings. Data were recorded over a total of 25 daily sessions in each monkey (monkey 1—17 sessions in experiment 1 and 8 sessions in experiment 2; monkey 2—10 sessions in experiment 1 and 15 sessions in experiment 2). The switch to experiment 2 was made after the animal showed a robust reward-prediction signal. Notably, electrodes were manually advanced by a minimum of $62.5\,\mathrm{\mu m}$ before every session to ensure that activity from new cells was recorded. Neural activity was amplified, filtered (300 Hz to 10 kHz) and stored for offline preprocessing and analysis. Cluster separation was applied (valley-seeking algorithm), and the binary spike train was smoothed using a Gaussian window ($\sigma =50\,\mathrm{ms}$). We collected spiking activity from 146 and 230 neurons in experiment 1 and from 205 and 151 neurons in experiment 2, for monkey 1 and monkey 2, respectively. Only cells sampled from the ventral and dorsal lateral frontal cortexes were included in the data (Extended Data Fig. 7a–c). No neurons were excluded based on their selectivity profiles. Notably, as the focus of this study was to track how learning influenced neural geometry and not the magnitude of firing (for example, repetition suppression effects), we *z* scored firing rates of each neuron across the whole session. The obtained firing-rate data were then epoched from 200 ms before to 1,200 ms after the color onset. Next, the full set of sessions in each animal were divided up into four learning stages and then sessions in each stage were pooled across animals, for example, the first learning stage was comprised of first five sessions from monkey 1 and first three sessions from monkey 2. We found that four learning stages were sufficient to capture learning-induced effects. To assess whether the choice of learning-stage discretization influenced the results in Fig. 4, we repeated the analyses using three to six learning stages. For all discretizations, each stage was required to include a minimum of six sessions, ensuring adequate statistical power. For the three-stage and four-stage schemes, sessions were grouped into approximately equal blocks. For the five-stage and six-stage schemes, a sliding-window approach was used (for example, stage 1 = sessions 1–6, stage 2 = sessions 5–10) to maintain comparable neuron counts and hence statistical power across discretizations. All analyses were implemented in Python using custom-written code and run on combined data (monkeys 1 and 2). Two types of analyses were used in this study—(1) time point-resolved method, where a specific method was applied to every time point in the epoch to track how representations evolved in trial time, and (2) time-averaged method, where a method was run on time-averaged data (for example, $\left({t}_{0\,\mathrm{ms}},{t}_{500\,\mathrm{ms}}\right)$, color-locked or shape-locked period) in the time window preceding the shape display or trial outcome. In the former time window, we examined the neural geometry when only the color information is known, whereas, just before outcome onset, we examined whether neural geometry reflected the color and the shape and their combination (XOR) before the animals received feedback about the value of the trial.

#### Adaptive trial termination and switch costs

To assess learning, we measured the proportion of trial terminations through fixation breaking in both rewarded and nonrewarded trials. Specifically, for each session, we counted the number of trial terminations in rewarded and nonrewarded trials when fixation breaking occurred during the shape-locked period (Fig. 2b), a phase where all necessary information for outcome prediction is available but the reward is not yet delivered. These counts were then normalized by dividing the total number of fixation errors recorded in the session. The adaptive trial termination measure was computed by dividing the normalized nonreward trial count by the normalized rewarded trial count for each session separately. We then divided sessions into four learning stages and fitted a linear regression model with learning stage as the predictor of the adaptive trial termination. To estimate the *P* value, we used a permutation approach, randomizing the session-to-learning stage association ($n=\mathrm{10,000}$ permutations). Switch costs were computed by comparing correct trial termination counts between color-switch and color-repeat trials; to isolate color-specific effects, shape-switch costs were subtracted from color-switch costs.

#### Existing lPFC dataset

We also used an existing dataset of electrophysiological recordings^21^ that have been described in detail previously^22,23^. In brief, neural activity was recorded from the ventral and dorsal lateral PFC (similar to the areas targeted in this study) in four rhesus monkeys who performed a feature match-to-sample task. More specifically, the animals were required to report, after a delay period, whether the shape of the first stimulus was the same as that of the second stimulus. Please note that a match/no-match rule is equivalent to an XOR rule. Notably, neural activity was recorded before the animals were exposed to the task rule (passive viewing) and after they had learned the rule. As both correct match and correct no-match trials were rewarded, the match/no-match signal was not confounded with a reward-prediction signal. To test whether neural activity was pushed toward a minimal regime in such experimental conditions, we used the same decoding and selectivity measures as used in the analysis of our dataset (Fig. 2). We examined neural data averaged across the presentation of the second stimulus and the subsequent delay period ($\left({t}_{0\,\mathrm{ms}},{t}_{2000\,\mathrm{ms}}\right)$; stimulus 2-locked period). Furthermore, neural activity was analyzed for all stimulus pairs combined. For the eight stimuli, we paired them into four sets of pairs and performed our analyses separately on each pair of stimuli (and averaged results over all four pairs) so that chance decoding was the same as in our dataset (that is, 50%).

### Models

#### Multiple linear regression

We can model the firing rate ${\boldsymbol{r}}$ of a neuron (either from our generative models or our data) at a given time point as a linear combination of the three main task variables—color, shape and the interaction between color and shape (the XOR term):

1 $${\bf{r}}=X{\boldsymbol{\beta }}+\epsilon$$

where **r** is a vector of $1\times \,K$ dimensionality containing the time-averaged firing rates for *K* trials; *X* is the design matrix of dimensionality *K* × *D* where rows correspond to the *K* trials and columns correspond to the value of the *D* task variables such as color, shape and XOR (*D* = 3) in each trial. **β** is a *D*-by-1 vector populated with the coefficients for each of the task variable estimated for the *n*th neuron. Finally, ${\boldsymbol{\epsilon }}$ contains *K* residuals. The **β** vector specifies the coordinates of the *n*th neuron in the selectivity space spanned by *D* task variables (Figs. 1b,c and 2k,l). That is, every neuron can be represented as a point in a space where each axis corresponds to the cell’s selectivity for a task variable. An equivalent linear model was used to characterize the firing rate for neurons in experiment 2 as a linear combination of the three variables context, stimulus set and the nonlinear mixture of context and stimulus set (XOR 2).

#### Generative models

Neural selectivity can be defined by the matrix ${\mathbf{S}}_{\mathrm{data}}=\left(S_{nd}\right)_{1\le n\le N,\,1\le d\le D}$, where each row n corresponds to a unit and each column d contains the regression coefficient for one task variable. In experiment 1, these variables were color, shape and their interaction (color × shape; XOR); in experiment 2, they were stimulus set, the interaction between stimulus set and context (XOR 2) and context. This cloud of points is then centered by removing the mean (${\sum }_{n}{S}_{nd}=0$, for each of the D task variables). Here we explored two types of selectivity distributions and their representational properties. First, we examined a random mixed selectivity model in which selectivities are captured by a spherical multivariate Gaussian distribution ${\mathbf{S}}_{\mathrm{random}}\,\sim\,\mathscr{N}_{D}\!\left(\mathbf{0},\,\sigma^{2}\mathbf{I}_{D}\right)$. In such a model, all variables can be decoded equally well from the population, resulting in a high-dimensional representation, and there is poor cross-generalization across variables. The second selectivity model we examine results from a system performing the task while being constrained to show low overall firing rates (that is, a form of metabolic cost). We derived analytically that maximizing XOR (experiment 1) or the context (experiment 2) decodability while minimizing such a metabolic cost results in units being selective only to the task-relevant variable and having no selectivity to the linear terms (color or shape; Supplementary Note). A matrix describing the selectivity of such a population can be thus formulated as ${\mathbf{S}}_{\mathrm{minimal}}\,\sim\,\mathscr{N}_{D}\left(\mathbf{0},\sigma^{2}\mathrm{diag}\left(0,0,1\right)\right)$, where the covariance matrix is a diagonal matrix with two first diagonal terms equal to zero and the third equal to one. We call this the minimal model. Notably, to allow comparisons between the observed selectivity and model selectivity (minimal or random), the generative models were constructed using parameters derived from the data. Specifically, we used the mean value of diagonal entries of the covariance matrix ${\widetilde{{\boldsymbol{\Sigma }}}}_{\mathrm{data}}$ estimated from $\mathbf{S}_{\mathrm{data}}$ to set the value of the variance parameter ${\sigma }^{2}$ in both ${\mathbf{S}}_{\mathrm{random}}$ and $\mathbf{S}_{\mathrm{minimal}}$ (please note that this ensures that both models have the same total variance). Furthermore, we showed that multiple linear regression was able to recover the underlying minimal and random models from artificially generated firing rates under various levels of noise (Extended Data Fig. 7d–g). To mimic measurement variability under finite sampling, we added zero-mean Gaussian noise ($\sigma \,=\,0.7$) to the generated selectivity coefficients (Fig. 1 and Extended Data Fig. 5b,c). Quantitative comparisons were performed between the observed neural selectivity coefficients and the corresponding idealized noise-free models (Extended Data Fig. 2a,b).

#### Optimized feedforward networks

We used n=400 units in these networks and their firing rates were described in equation (1) with σ = 2. The output ${\boldsymbol{z}}$ of these networks was given by a softmax readout

2 $$z=\mathrm{Softmax}\left({W}_{\mathrm{out}}{\bf{r}}+b\right)$$

where *W*_out_ are the two sets of readout weights (connecting the hidden layer to the readout unit 1 (XOR == 0) and weights connecting the hidden layer to readout unit 2 (XOR == 1)) and *b* is the readout bias. We optimized these networks with backpropagation using a canonical cross-entropy cost function

3 $${L}={H}(p,z)+\frac{\lambda }{2n}{||{\bf{r}}||}_{2}^{2}$$

where the first part of equation (3) denotes the cross-entropy loss ${H}(p,z)$ between the true probabilities of reward p (which were equal to 0 or 1, depending upon the stimuli for that trial) and the model’s readout probabilities ${\boldsymbol{z}}$, and the second term corresponds to a metabolic cost on all firing rates. Before training, the values of the **β**s were drawn randomly from a Gaussian distribution with 0 mean and variance 0.05, and elements of *W*_out_ and *b* were set to 0. We trained the following two types of models: (1) with no regularization (λ=0; Extended Data Fig. 1a) and (2) with high regularization (λ=20; Extended Data Fig. 1b). In line with our predictions, selectivity to task variables in models trained with high regularization converged on the minimal selectivity regime, whereas models trained with no regularization produced randomly mixed selectivity (Extended Data Fig. 1c,d). This network formulation maps directly onto the selectivity space examined in experiment 2—stimulus set, stimulus set × context and context—with context as the task-relevant variable as the XOR was not explicitly solved within the network but was provided as an input feature, differing from other inputs only in its relevance to the output.

### Analysis methods

#### Decoding

To test which information was represented by the observed neural population as a function of learning, we used linear SVM decoding^8,16^. In contrast to the regression analysis, where the regression coefficients were estimated for every neuron separately, decoding analyses were run on pseudopopulations. This approach enhanced statistical power, reducing the likelihood of type II errors, and mitigated the impact of session-specific sampling bias. Given the varying number of trials per session (both within and between animals), a sliding-window method was used to apply all available data. Specifically, each session was divided into three windows, each matching the size of the session with the fewest trials (n=801). Neural activities ($\mathrm{trials}\times \mathrm{neuron}\times \mathrm{time}$) for the first 801 trials in each session during learning stage 1 were combined along the neuron axis to form a pseudopopulation. This procedure was repeated for the middle and final 801 trials across each learning stage. This resulted in a matrix $\mathbf X={\left({X}_{knt}\right)}_{1\le k\le K,1\le n\le {n}_{\mathrm{neuron}},1\le t\le T}$ for each of the time windows and each of the four learning stages, where the first dimension corresponds to *K* trials, the second dimension corresponds to *n*_neuron_ (combined from two animals), and the last dimension corresponds to *T* time points. Then binary SVM classifiers were used to decode the task variables (color, shape, width and XOR in experiment 1 and context, stimulus set, stimulus set × context (XOR 2), shape, width and XOR in experiment 2) at each time point for temporally resolved decoding (Figs. 2e,f,g,i, 3e,f,g and 4d,h,l and Extended Data Figs. 3i–l and 6a–c). The statistical tests were run on firing rates average in broad time windows covering the entire feature presentation period (denoted by the pale orange shaded areas). An equivalent decoding procedure was used when analyzing generative models (Extended Data Fig. 2e). Decoding was performed in a cross-validated manner, in which *K* trials were randomly split into set 1 and set 2, with each set containing 50% of the trials. The decoder was fitted using set 1 and tested on set 2. The procedure was then repeated using set 2 as the training set and set 1 as the test set. Both decoding scores were then averaged. This procedure was repeated ten times for different random splits of trials in sets 1 and 2, and these ten resulting scores were then averaged. The decoding results were averaged over three time windows.

#### Shattering dimensionality

**Extended Data Fig. 8:**
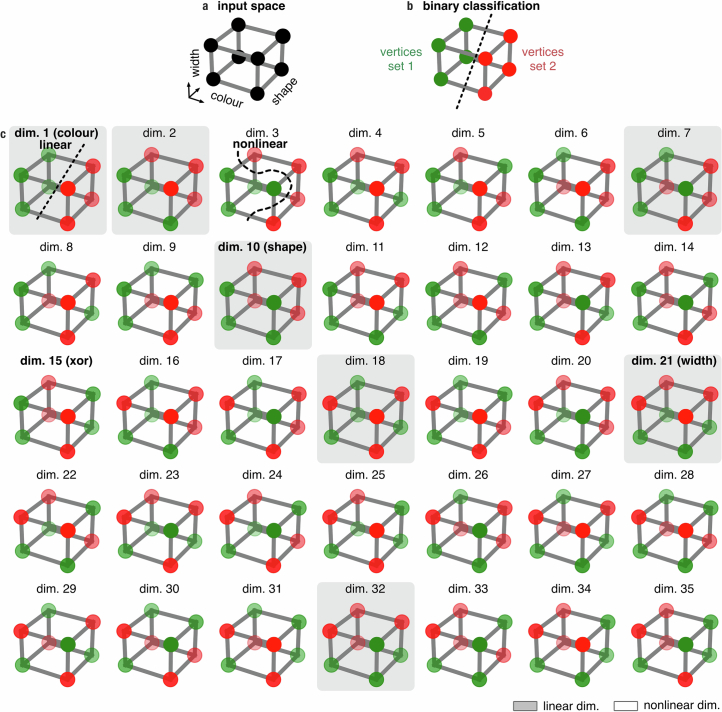
Schematic depiction of shattering dimensionality in the XOR task in experiment 1. **a**, Color, shape, and width form a cube in the input space, with each of the 8 vertices representing a unique combination of these input variables. **b**, The vertices can be divided into two equally sized groups of four vertices each, representing a binary classification problem. **c**, All 35 theoretically possible binary problems (red vs. green vertices) are obtained by randomly splitting the vertices into two equally sized groups. Dimensions 1, 10, 15, and 21 correspond to color, shape, XOR, and width, respectively. The gray and white backgrounds indicate whether a dimension is linear (can be split by a plane, for example, dimension 1) or nonlinear (cannot be split by a plane, for example, dimension 3), respectively. Color intensity corresponds to spatial depth, with solid colors signifying vertices closer to the viewer and pale colors signifying those farther away.

To estimate shattering dimensionality^8,16^ in experiment 1 (Fig. 2j), we used the same decoding approach as described above except that we averaged decoding scores over all 35 possible dichotomies that could be theoretically represented given the task structure (that is, three linear variables form a cube in state space that can be dissected into two sets of four vertices in 35 possible ways; see Extended Data Fig. 8 for illustration). We estimated the decodability of each dichotomy and tracked the mean decoding accuracy for each as a function of learning. Exploring the theoretically maximal dimensionality of the color-locked neural representations in experiment 2 using shattered dimensionality, where two linear variables (color and stimulus set) were used, resulted in the identification of three (color, stimulus set, color × stimulus set (XOR 2)) theoretically possible binary decoding problems. We tracked the mean of these dimensions over time (Fig. 3h).

#### Cross-generalized decoding

**Extended Data Fig. 9:**
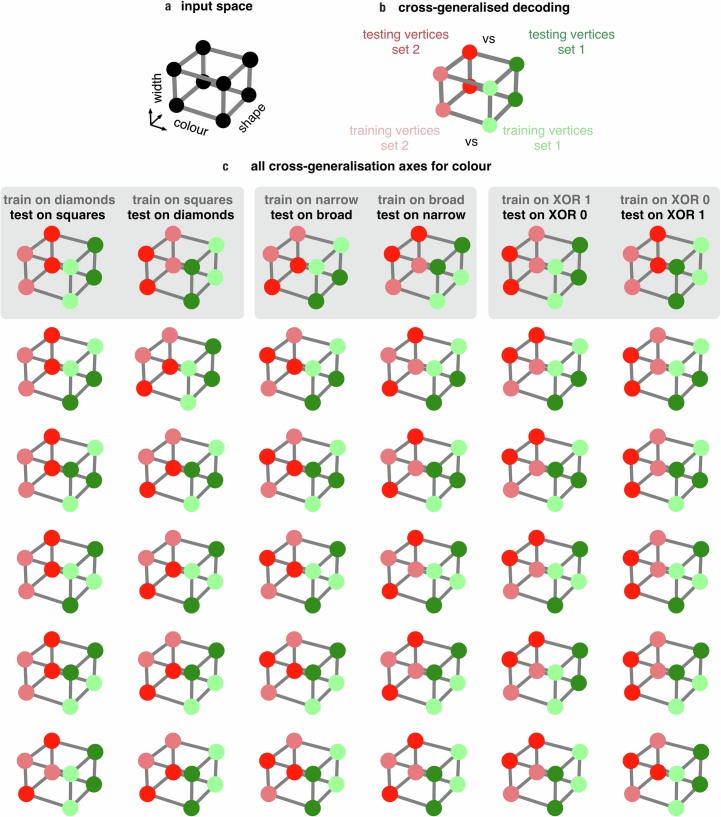
Schematic depiction of cross-generalized decoding of the variables. **a**, Color (blue vs green), shape (diamond vs square), and width (narrow vs broad) form a cube in the input space, with each of the 8 vertices representing a unique combination of these input variables. **b**, To test whether a variable is encoded in an abstract format relative to a single variable, two binary linear classification problems were defined. First, a classifier was trained on differentiating that variable (for example, color) only on a subset of trials (for example, diamond shape trials). Next, this classifier was tested on the remaining subset of trials (for example, square shape trials). For example, a high score obtained from such a decoding procedure indicates that the same representation of color was used as a function of different levels of shape, a hallmark of abstract coding. **c**, To test whether a variable (for example, color) has an abstract format relative to all remaining task variables, this procedure needs to be repeated for all possible train and test splits (when color 1 is always on the left and color 2 is always on the right side of the cube). These 36 scores are then averaged to obtain cross-generalized decoding of color. Some of the test-train splits correspond to task variables like shape, width and XOR (gray background) while others represent a mixture of input variables.

To examine the neural geometry of the task variables, we used cross-generalized SVM decoding^16^. In contrast to a typical cross-validation procedure, the testing happens not only on trials that were previously not seen but also on trials that correspond to different conditions. To achieve a high cross-generalization score, it is therefore not sufficient to generalize across trial-wise noise, but also to generalize across conditions^16^. Specifically, the labels of eight unique conditions ($\mathrm{two}\,\mathrm{colors}\times \mathrm{two}\,\mathrm{shapes}\times \mathrm{two}\,\mathrm{widths}$; Extended Data Fig. 9a) were split into two sets of four labels each, depending on the tested variable (for example, color 1 labels versus color 2 labels; Extended Data Fig. 9b). Each subset was further divided into a training set and a testing set (color 1 versus color 2 training set and color 1 versus color 2 testing set). A decoder was trained on the training set and then tested on the testing set, and vice versa; the two scores were then averaged. We identified 36 possible train-test splits (Extended Data Fig. 9c), and the cross-generalized decoding score was obtained by averaging these scores. This method was used to determine whether the format of a task variable is abstract, meaning the variable is encoded in the same format as a function of the remaining task variables. Please note that some of the train-test splits correspond to decoding a task variable as a function of a single other variable (for example, decoding color as a function of shape; Extended Data Fig. 9c, shaded geometries), while others examine the decoding of the variable as a function of a mix of variables.

#### Cross-stimulus set generalization (decoding and selectivity analyses)

Cross-generalized decoding performed for experiment 2 data (both run on time-resolved and time-averaged firing rates in Fig. 4c,g,k and Fig. 4d,h,l, respectively) differed in one aspect from the algorithm described in Cross-generalized decoding. As the aim of the analysis described here was to identify the neural format of the main task variables used across stimulus sets, only one splitting variable was used (that is, stimulus set) to obtain cross-generalization scores for the task-relevant variables (context, shape and XOR). This reduced the possible cross-generalization decoding axes to four possible binary decoding problems (for example, when performing cross-generalized decoding for the color variable we can (1) train on differentiating color 1 from color 2 in stimulus set 1 and test on differentiating color 3 from color 4 in stimulus set 2, (2) train on differentiating color 3 from color 4 in stimulus set 2 and test on differentiating color 1 from color 2 in stimulus set 1, (3) train on differentiating color 1 from color 3 and test on differentiating color 2 from color 4, and (4) train on differentiating color 2 from color 4 and test on differentiating color 1 from color 3; these four decoding scores were then averaged). Using this procedure, we explored the cross-stimulus set generalization potential of the color, shape, width and XOR variables. Additionally, to test how selectivity of PFC cells changed as a function of learning in experiment 2 we used a Pearson correlation metric. Specifically, we compared how similar the color, shape and XOR coefficients in stimulus set 1 are to coefficients for the same variables in stimulus set 2 (Fig. 4a,e,i), which yielded three correlation scores for each of the main task variables. This was done for each of the four learning stages to explore whether selectivity for stimulus set 1 aligns with that for stimulus set 2 as a function of learning, consistent with a shared abstract representation (Fig. 4b,f,j).

#### Measuring similarity between selectivity distributions

**Extended Data Fig. 10:**
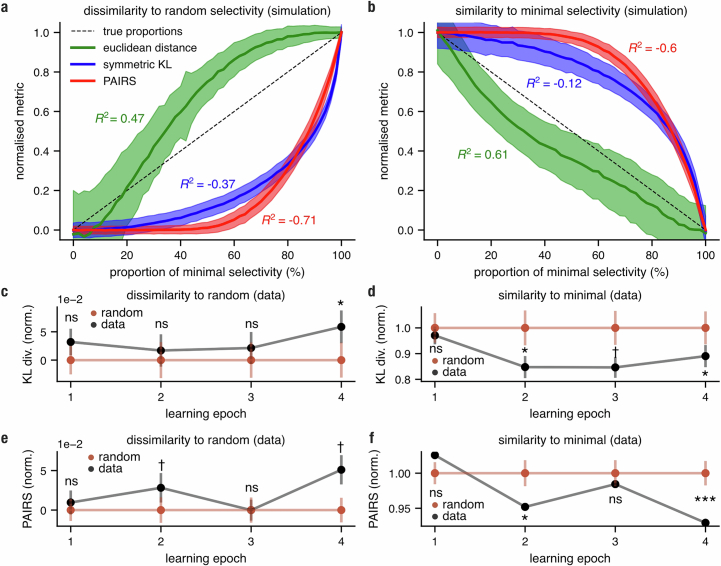
The precision of Euclidean distance, symmetric KL estimate and PAIRs metrics in tracking learning-induced changes to neural selectivity. **a**, Relative Euclidean distance between the covariance matrix of selectivity coefficients obtained from a simulated mixed population (random-minimal) and the covariance matrix expected from pure random selectivity plotted as a function of minimal selectivity proportions (0–100%; for details, see Measuring similarity between selectivity distributions). Colored annotations indicate mean R^2^ values computed between the true proportions (dotted lines) and estimated proportions (colored bold lines). **b**, Same as **a**, but the relative distance from the covariance matrix expected from minimal selectivity is shown; shaded areas illustrate mean $\pm 1{\rm{s}}.{\rm{d}}.$ for each of the metrics computed from 1,000 randomly drawn selectivity models. **c**,**d**, Selectivity results from experiment 1 (Fig. 2m,n) computed using symmetric KL divergence estimate. **e**,**f**, Selectivity results from experiment 1 (Fig. 2m,n) computed using the PAIRs metric.

To test the observed neural population for the presence of random mixed or minimal selectivity (Figs. 1b,c and 2k,l), we first obtained regression coefficients for the three variables of interest (color, shape and XOR; equation (1)) and then constructed the selectivity space *S*_data_. To assess the similarity of *S*_data_ to *S*_minimal_ and *S*_random_, we computed the covariance matrix of *S*_data_ (${\widetilde{{\boldsymbol{\Sigma }}}}_{\mathrm{data}}$) as well as the covariance matrices of the expected random and minimal distributions given *S*_data_ (${\widetilde{{\boldsymbol{\Sigma }}}}_{\mathrm{minimal}}$ and ${\widetilde{{\boldsymbol{\Sigma }}}}_{\mathrm{random}}$; ‘Generative models’). Finally, we calculated the normalized distance of the observed selectivity from model random selectivity

4 $${d}_{i\,\mathrm{from}\,\mathrm{random}}\left(\widetilde{{\boldsymbol{\Sigma }}}\right)=\frac{{\Vert \widetilde{{\boldsymbol{\Sigma }}}-{\widetilde{{\boldsymbol{\Sigma }}}}_{i\,\mathrm{random}}\Vert }_{2}-{\mathbb{E}}\left({\Vert {\widetilde{{\boldsymbol{\Sigma }}}}_{i\,\mathrm{random}}-{\widetilde{{\boldsymbol{\Sigma }}}}_{i\,\mathrm{random}}\Vert }_{2}\right)}{{\mathbb{E}}\left({\Vert {\widetilde{{\boldsymbol{\Sigma }}}}_{i\,\mathrm{random}}-{\widetilde{{\boldsymbol{\Sigma }}}}_{i\,\mathrm{minimal}}\Vert }_{2}\right)-{\mathbb{E}}\left({\Vert {\widetilde{{\boldsymbol{\Sigma }}}}_{i\,\mathrm{random}}-{\widetilde{{\boldsymbol{\Sigma }}}}_{i\,\mathrm{random}}\Vert }_{2}\right)}$$

and the normalized distance of the observed selectivity to minimal selectivity

5 $${d}_{i\,\mathrm{from}\,\mathrm{minimal}}(\widetilde{{\boldsymbol{\Sigma }}})=\frac{{\Vert \widetilde{{\boldsymbol{\Sigma }}}-{\widetilde{{\boldsymbol{\Sigma }}}}_{i\,\mathrm{minimal}}\Vert }_{2}-{\mathbb{E}}\left({\Vert {\widetilde{{\boldsymbol{\Sigma }}}}_{i\,\mathrm{minimal}}-{\widetilde{{\boldsymbol{\Sigma }}}}_{i\,\mathrm{minimal}}\Vert }_{2}\right)}{{\mathbb{E}}\left({\Vert {\widetilde{{\boldsymbol{\Sigma }}}}_{i\,\mathrm{random}}-{\widetilde{{\boldsymbol{\Sigma }}}}_{i\,\mathrm{minimal}}\Vert }_{2}\right)-{\mathbb{E}}\left({\Vert {\widetilde{{\boldsymbol{\Sigma }}}}_{i\,\mathrm{minimal}}-{\widetilde{{\boldsymbol{\Sigma }}}}_{i\,\mathrm{minimal}}\Vert }_{2}\right)}$$

where the subscript *i* denotes a random draw and the expectations were computed over 1,000 draws. From both the numerators and denominators, the distance within each model was subtracted to center the measure around 0. More specifically, ${\mathbb{E}}\left({\Vert {\widetilde{{\boldsymbol{\Sigma }}}}_{i\,\mathrm{random}}-{\widetilde{{\boldsymbol{\Sigma }}}}_{i\,\mathrm{random}}\Vert }_{2}\right)$ (the expected difference between two different randomly drawn selectivity distributions from the random model) was, for example, subtracted from the denominator and numerator of ${d}_{\mathrm{from}\,\mathrm{random}}$ to account for within-model distance. Additionally, both ${d}_{\mathrm{from}\,\mathrm{random}}$ and ${d}_{\mathrm{from}\,\mathrm{minimal}}$ were normalized by the distance between selectivities generated using both generative models $\left({\mathbb{E}}\left({\Vert {\widetilde{{\boldsymbol{\Sigma }}}}_{i\,\mathrm{random}}-{\widetilde{{\boldsymbol{\Sigma }}}}_{i\,\mathrm{minimal}}\Vert }_{2}\right)\right)$ that resulted in the metrics being bounded between 0 and 1 (when ${\Vert {\widetilde{{\boldsymbol{\Sigma }}}}_{\mathrm{data}}-{\widetilde{{\boldsymbol{\Sigma }}}}_{i\,\mathrm{minimal}}\Vert }_{2}$ is equal or smaller than ${\Vert {\widetilde{{\boldsymbol{\Sigma }}}}_{i\,\mathrm{random}}-{\widetilde{{\boldsymbol{\Sigma }}}}_{i\,\mathrm{minimal}}\Vert }_{2}$). This was done to allow for a comparison of similarity estimates across the learning stages. The Euclidean distance metric was chosen as the main analysis tool in this study based on simulations in which we generated different proportions of random and minimal selectivity across a single population (from 0% minimal and 100% random to 100% minimal and 0% random) and compared the precision with which multiple metrics recovered the true proportions. We compared the Euclidean distance metric to the PAIRs metric, which has been used previously in the literature^5,24^, and to the symmetric Kullback–Leibler divergence (KL divergence) estimate that benefits from a strong theoretical basis and is assumption agnostic. We found that, compared to the KL divergence and PAIRs metrics, the Euclidean distance measure yielded the highest precision of tracking learning-induced changes to neural selectivity (Extended Data Fig. 10a,b). Specifically, our simulations showed that both the KL divergence and PAIRs can be used to precisely identify extreme selectivity regimes (either strong random selectivity or strong minimal selectivity) but fail at identifying intermediate selectivity regimes that show a strong bias toward random mixed selectivity (Extended Data Fig. 10a,b). As the focus of this study was to track learning dynamics, a metric that identifies a broad range of selectivity regimes was chosen for the final analysis. Nonetheless, the results from experiment 1 (Fig. 2m,n) were broadly replicated using the symmetric KL divergence estimate and PAIRs (Extended Data Fig. 10c–f).

#### PCA

PCA was used as a measure of neural dimensionality in experiment 1 (Extended Data Fig. 3h). First, pseudopopulations were constructed for each learning stage using the same procedure as described in Decoding. Then, firing rates were averaged in the time window preceding the outcome presentation ($\left({t}_{400\,\mathrm{ms}},{t}_{500\,\mathrm{ms}}\right)$, shape-locked period). Next, PCs were run on condition averages. This was done separately for each learning stage. To compute how the variance explained (ratio) by the first PC changed as a function of learning, trials were randomly split into test and train ten times; PCA was fitted then on train trials and the test trial firing rates were projected onto them to compute variance explained. The results from ten random splits were then averaged. Please note that width 1 and width 2 trials were pooled together. The null distribution for the permutation test was computed by randomly shuffling neurons between stage 1 and stage 4, and repeating the described PCA procedure (n=500).

### Statistical testing and reproducibility

#### Decoding and cross-generalized decoding

Throughout the study, we used nonparametric permutation tests to test statistical significance within each learning stage and between learning stages (learning-induced effects). Two types of null distributions were thus constructed—(1) for statistical testing of above chance-level decoding analyses, the labels describing the trial dimension (k) of the pseudopopulation matrix $\mathbf X={({X}_{{knt}})}_{1\le k\le K,1\le n\le N,1\le t\le T}$ were randomly permuted 1,000 times; and (2) to test for learning-induced effects on decoding scores, the matrices $X_{\mathrm{stage}-1}$ and $X_{\mathrm{stage}-4}$ were concatenated along the neuron dimension (n) and then 1,000 new ${\mathbf X{{\prime} }}_{\mathrm{stage}-1}$ and ${{ \mathbf X}{{\prime} }}_{\mathrm{stage}-4}$ matrices were generated by randomly assigning neurons to either ${{ \mathbf X}{{\prime} }}_{\mathrm{stage}\,1}$ or ${{\mathbf X}{{\prime} }}_{\mathrm{stage}\,4}$. One-sided tests were used when testing the predictions of the minimal model and two-sided tests were used when no differences were expected.

#### Selectivity measures

To test whether observed selectivity was dissimilar to the random selectivity regime and similar to minimal selectivity regime, 1,000 random and minimal models were generated using data-derived parameters for each learning stage. Next, the ${d}_{\mathrm{rand}.\mathrm{fromrand}.}$ and ${d}_{\mathrm{rand}.\mathrm{frommin}.}$ distances were computed for 1,000 randomly generated models according to equations (4) and (5) (with ${\widetilde{{\boldsymbol{\Sigma }}}}_{\mathrm{random}}$ as input) to serve as null distributions for both comparisons. Please note that the observed selectivity was compared to random model selectivity when analyzing the data’s similarity to random (Figs. 2m and 3m) as well as minimal selectivity (Figs. 2n and 3n). Furthermore, as in experiment 2 we tested whether selectivity for task variables was similar in stimulus set 1 to variables in stimulus set 2 and whether this selectivity alignment changed over learning, two null distributions were thus constructed—(1) statistically significant selectivity alignment was assessed by comparing the observed correlation to a distribution (n=1,000) of correlations obtained after shuffling one of the selectivity vectors; and (2) learning-induced effects in selectivity alignment were assessed by comparing the observed difference in alignment between stage 1 and stage 4 to a distribution of differences computed after randomly shuffling neurons between stage 1 and stage 4.

### Reporting summary

Further information on research design is available in the Nature Portfolio Reporting Summary linked to this article.

## Online content

Any methods, additional references, Nature Portfolio reporting summaries, source data, extended data, supplementary information, acknowledgements, peer review information; details of author contributions and competing interests; and statements of data and code availability are available at 10.1038/s41593-026-02333-w.

## Supplementary information

Supplementary Information Supplementary Note.

Reporting Summary

## Source data

Source Data Fig. 2 Statistical source data.

Source Data Fig. 3 Statistical source data.

Source Data Fig. 4 Statistical source data.

Source Data Extended Data Fig. 1 Statistical source data.

Source Data Extended Data Fig. 2 Statistical source data.

Source Data Extended Data Fig. 3 Statistical source data.

Source Data Extended Data Fig. 4 Statistical source data.

Source Data Extended Data Fig. 5 Statistical source data.

Source Data Extended Data Fig. 6 Statistical source data.

Source Data Extended Data Fig. 7 Statistical source data.

## Data Availability

The dataset supporting the findings of this study is accessible through Dryad^47^. For any additional details, please contact the corresponding author. Source data are provided with this paper.

## Code availability

All code for this study was custom written in Python using the NumPy, SciPy, Matplotlib, Scikit-learn and TensorFlow libraries. The code repository is publicly available on GitHub (https://github.com/m-j-wojcik/pfc_learning).
