# Supplementary material for: Learning shapes neural geometry in the primate prefrontal cortex
Source: Nat Neurosci. 2026 Jun 25;29(8):1966–75. doi: 10.1038/s41593-026-02333-w (PMC13433252; doi:10.1038/s41593-026-02333-w)
Supplement: Supplementary file 1 [file 41593_2026_2333_MOESM1_ESM.pdf]

nature neuroscience

Supplementary information https://doi.org/10.1038/s41593-026-02333-w

Learning shapes neural geometry in the primate prefrontal cortex

In the format provided by the authors and unedited

Learning shapes neural geometry in the primate prefrontal cortex

Supplementary information

Section 1.1: How to set selectivity parameters for optimal XOR decoding

We assume that neural activities $\boldsymbol{x}$ of $\boldsymbol{N}$ neurons are given by the following regression model

$$\boldsymbol{x} = \boldsymbol{\beta}_0 + \delta_{c,c_1} \boldsymbol{\beta}_C + \delta_{s,s_1} \boldsymbol{\beta}_S + \delta_{c,c_1} \delta_{s,s_1} \boldsymbol{\beta}_I + \boldsymbol{\eta} \tag{1}$$

Where $\boldsymbol{\beta}_C , \boldsymbol{\beta}_S , \boldsymbol{\beta}_I$ are the regression 'coefficients' for colour, shape, and the interaction term, respectively, $\delta_{c,c_1} = 1$ if the colour c is colour 1 and -1 otherwise (same for δ_{s,s_1} for shapes s), and $\boldsymbol{\eta} \sim \mathcal{N}(\boldsymbol{0}, \boldsymbol{\Sigma}_1)$. Therefore,

$$\boldsymbol{x}|r_1 \sim \mathcal{N}(\boldsymbol{\mu}_{r_1}, \boldsymbol{\Sigma}_1) \tag{2}$$

$$\boldsymbol{x}|r_2 \sim \mathcal{N}(\boldsymbol{\mu}_{r_2}, \boldsymbol{\Sigma}_2). \tag{3}$$

Where r_1 is one XOR condition (i.e., $c = 1, s = 1$ or $c = 2, s = 2$) and r_2 is the other XOR condition (i.e., $c = 1, s = 2$ or $c = 2, s = 1$) and $\boldsymbol{\Sigma}_2$ is some noise covariance matrix. Note that with the inclusion of the interaction term, it is sufficient to separate the two XOR conditions. We now calculate $\boldsymbol{\mu}_{r_1}$ and $\boldsymbol{\mu}_{r_2}$:

$$\begin{aligned}\boldsymbol{\mu}_{r_2} &= \frac{\boldsymbol{\beta}_0 + \boldsymbol{\beta}_C + \boldsymbol{\beta}_S + \boldsymbol{\beta}_I}{2} + \frac{\boldsymbol{\beta}_0 - \boldsymbol{\beta}_C - \boldsymbol{\beta}_S + \boldsymbol{\beta}_I}{2} \\ &= \boldsymbol{\beta}_0 + \boldsymbol{\beta}_I\end{aligned} \tag{4}$$

and,

$$\begin{aligned}\boldsymbol{\mu}_{r_2} &= \frac{\boldsymbol{\beta}_0 - \boldsymbol{\beta}_C + \boldsymbol{\beta}_S - \boldsymbol{\beta}_I}{2} + \frac{\boldsymbol{\beta}_0 + \boldsymbol{\beta}_C - \boldsymbol{\beta}_S - \boldsymbol{\beta}_I}{2} \\ &= \boldsymbol{\beta}_0 - \boldsymbol{\beta}_I\end{aligned} \tag{5}$$

Therefore,

$$\boldsymbol{x}|r_1 \sim \mathcal{N}(\boldsymbol{\beta}_0 + \boldsymbol{\beta}_I, \boldsymbol{\Sigma}_2) \tag{6}$$

$$\boldsymbol{x}|r_2 \sim \mathcal{N}(\boldsymbol{\beta}_0 - \boldsymbol{\beta}_I, \boldsymbol{\Sigma}_2). \tag{7}$$

Therefore, we need $\boldsymbol{\beta}_I > 0$ to be able to separate the two XOR conditions.

Section 1.2: An energy cost on unnecessary neural activity

If we also consider minimising the squared norm of neural activity for each condition (i.e., an energy cost), we have

$$\mathbb{E}\left[\left\|\boldsymbol{x}_{c,s}\right\|^2\right] = \mathbb{E}\left[\boldsymbol{x}_{c,s}\right]^{\mathrm{T}} \mathbb{E}\left[\boldsymbol{x}_{c,s}\right] + \mathrm{Tr}(\boldsymbol{\Sigma}_1) \tag{8}$$

where the subscripts c and s correspond to colour and shape indices, respectively. Therefore,

$$\mathbb{E}\left[\left\|\boldsymbol{x}_{1,2}\right\|^2\right] = (\boldsymbol{\beta}_0 - \boldsymbol{\beta}_C + \boldsymbol{\beta}_S - \boldsymbol{\beta}_I)^{\mathrm{T}} (\boldsymbol{\beta}_0 - \boldsymbol{\beta}_C + \boldsymbol{\beta}_S - \boldsymbol{\beta}_I) + \mathrm{Tr}(\boldsymbol{\Sigma}_1) \tag{9}$$

$$\mathbb{E}\left[\left\|\boldsymbol{x}_{2,1}\right\|^2\right] = (\boldsymbol{\beta}_0 + \boldsymbol{\beta}_C - \boldsymbol{\beta}_S - \boldsymbol{\beta}_I)^{\mathrm{T}} (\boldsymbol{\beta}_0 + \boldsymbol{\beta}_C - \boldsymbol{\beta}_S - \boldsymbol{\beta}_I) + \mathrm{Tr}(\boldsymbol{\Sigma}_1) \tag{10}$$

$$\mathbb{E}\left[\left\|\boldsymbol{x}_{1,1}\right\|^2\right] = (\boldsymbol{\beta}_0 - \boldsymbol{\beta}_C - \boldsymbol{\beta}_S + \boldsymbol{\beta}_I)^{\mathrm{T}} (\boldsymbol{\beta}_0 - \boldsymbol{\beta}_C - \boldsymbol{\beta}_S + \boldsymbol{\beta}_I) + \mathrm{Tr}(\boldsymbol{\Sigma}_1) \tag{11}$$

$$\mathbb{E}\left[\left\|\boldsymbol{x}_{2,2}\right\|^2\right] = (\boldsymbol{\beta}_0 + \boldsymbol{\beta}_C + \boldsymbol{\beta}_S + \boldsymbol{\beta}_I)^{\mathrm{T}} (\boldsymbol{\beta}_0 + \boldsymbol{\beta}_C + \boldsymbol{\beta}_S + \boldsymbol{\beta}_I) + \mathrm{Tr}(\boldsymbol{\Sigma}_1) \tag{12}$$

Therefore, the total mean energy cost m is given by

$$m = \frac{1}{4} \sum_{c=1,2} \sum_{s=1,2} \mathbb{E}\left[\left\|\boldsymbol{x}_{c,s}\right\|^2\right] \tag{13}$$

$$= \boldsymbol{\beta}_0^{\mathrm{T}}\boldsymbol{\beta}_0 + \boldsymbol{\beta}_C^{\mathrm{T}}\boldsymbol{\beta}_C + \boldsymbol{\beta}_S^{\mathrm{T}}\boldsymbol{\beta}_S + \boldsymbol{\beta}_I^{\mathrm{T}}\boldsymbol{\beta}_I + \mathrm{Tr}(\boldsymbol{\Sigma}_1) \tag{14}$$

$$= \|\boldsymbol{\beta}_0\|^2 + \|\boldsymbol{\beta}_C\|^2 + \|\boldsymbol{\beta}_S\|^2 + \|\boldsymbol{\beta}_I\|^2 + \mathrm{Tr}(\boldsymbol{\Sigma}_1) \tag{15}$$

To minimise m while keeping $\boldsymbol{\beta}_I > 0$, which we need for performance, we can set $\boldsymbol{\beta}_0 = \boldsymbol{\beta}_C = \boldsymbol{\beta}_S = \mathbf{0}$ which gives

$$m = \|\boldsymbol{\beta}_I\|^2 + \mathrm{Tr}(\boldsymbol{\Sigma}_1) \tag{16}$$
